# Semimechanistic Pharmacokinetic and Pharmacodynamic Modeling of Piperaquine in a Volunteer Infection Study with Plasmodium falciparum Blood-Stage Malaria

**DOI:** 10.1128/AAC.01583-20

**Published:** 2021-03-18

**Authors:** Thanaporn Wattanakul, Mark Baker, Joerg Mohrle, Brett McWhinney, Richard M. Hoglund, James S. McCarthy, Joel Tarning

**Affiliations:** a Mahidol Oxford Tropical Medicine Research Unit, Faculty of Tropical Medicine, Mahidol University, Bangkok, Thailand; b Department of Clinical Pharmacology, ViiV Healthcare, Geneva, Switzerland; c Medicine for Malaria Venture, Meyrin, Switzerland; d Analytical Chemistry Unit, Pathology Queensland, Royal Brisbane and Women’s Hospital, Brisbane, QLD, Australia; e Centre for Tropical Medicine and Global Health, Nuffield Department of Medicine, University of Oxford, Oxford, United Kingdom; f QIMR Berghofer Medical Research Institute, Brisbane, QLD, Australia; g School of Medicine, The University of Queensland, Brisbane, QLD, Australia

**Keywords:** antimalarial agents, pharmacokinetics, pharmacodynamics, population pharmacokinetics, pharmacology, piperaquine, *P. falciparum* malaria, controlled human malaria infection, induced blood-stage malaria

## Abstract

Dihydroartemisinin-piperaquine is a recommended first-line artemisinin combination therapy for Plasmodium falciparum malaria. Piperaquine is also under consideration for other antimalarial combination therapies. The aim of this study was to develop a pharmacokinetic-pharmacodynamic model that might be useful when optimizing the use of piperaquine in new antimalarial combination therapies. The pharmacokinetic-pharmacodynamic model was developed using data from a previously reported dose-ranging study where 24 healthy volunteers were inoculated with 1,800 blood-stage Plasmodium falciparum parasites. All volunteers received a single oral dose of piperaquine (960 mg, 640 mg, or 480 mg) on day 7 or day 8 after parasite inoculation in separate cohorts. Parasite densities were measured by quantitative PCR (qPCR), and piperaquine levels were measured in plasma samples. We used nonlinear mixed-effect modeling to characterize the pharmacokinetic properties of piperaquine and the parasite dynamics associated with piperaquine exposure. The pharmacokinetics of piperaquine was described by a three-compartment disposition model. A semimechanistic parasite dynamics model was developed to explain the maturation of parasites, sequestration of mature parasites, synchronicity of infections, and multiplication of parasites, as seen in natural clinical infections with P. falciparum malaria. Piperaquine-associated parasite killing was estimated using a maximum effect (*E*_max_) function. Treatment simulations (i.e., 3-day oral dosing of dihydroartemisinin-piperaquine) indicated that to be able to combat multidrug-resistant infections, an ideal additional drug in a new antimalarial triple-combination therapy should have a parasite reduction ratio of ≥10^2^ per life cycle (38.8 h) with a duration of action of ≥2 weeks. The semimechanistic pharmacokinetic-pharmacodynamic model described here offers the potential to be a valuable tool for assessing and optimizing current and new antimalarial drug combination therapies containing piperaquine and the impact of these therapies on killing multidrug-resistant infections. (This study has been registered in the Australian and New Zealand Clinical Trials Registry under no. ANZCTRN12613000565741.)

## Abstract (precis)

Dihydroartemisinin-piperaquine is a recommended first-line artemisinin combination therapy for Plasmodium falciparum malaria. Piperaquine is also under consideration for other antimalarial combination therapies.

## TEXT

Dihydroartemisinin-piperaquine is one of the recommended first-line artemisinin-based combination therapies (ACTs) for uncomplicated Plasmodium falciparum malaria. In this antimalarial combination therapy, dihydroartemisinin serves as the rapid-acting component and piperaquine as a long-acting partner drug. The ongoing emergence of resistance to both artemisinin and its partner drugs is threatening malaria control and eradication (1). The presence of resistance to artemisinin alone is considered a partial resistance because parasites remain sensitive to the partner drug, resulting in cure. However, in this circumstance, artemisinin resistance requires the partner drug to clear a higher residual parasite biomass, which risks the emergence of resistance to the partner drug. Therefore, artemisinin resistance contributes to the emergence of multidrug-resistant parasites and to increased rates of treatment failure.

Resistance to artemisinin derivatives has been reported in the Southeast Asia region, i.e., Cambodia, Lao People’s Democratic Republic, Thailand, Myanmar, and Vietnam (1–3). Additionally, in 2016, a multisite prospective cohort study in Cambodia (4) demonstrated that patients with recrudescence presented with parasites with significantly decreased piperaquine susceptibility compared with patients without recrudescence (mean piperaquine 50% inhibitory concentration [IC_50_], 64.0 versus 21.4 ng/ml; *P* = 0.0002). In a separate study, a genome-wide association analysis of Plasmodium falciparum isolates from Cambodia demonstrated that amplification of genetic markers of piperaquine resistance, such as *exo-E415G* SNP, *plasmepsin 2*, and *plasmepsin 3*, was significantly associated with decreased treatment efficacy (5). Therefore, artesunate plus mefloquine has been substituted as a new first-line ACT in some Cambodian provinces (5). To counteract the emergence of drug resistance, clinical trials have been undertaken (6) to assess the efficacy of triple ACTs, such as dihydroartemisinin-piperaquine plus mefloquine, artemether-lumefantrine plus amodiaquine, and arterolane-piperaquine plus mefloquine. Additionally, a combination of the novel ozonide antimalarial artefenomel with piperaquine is under investigation (7).

Piperaquine is a 4-aminoquinoline antimalarial drug whose clinical pharmacology is characterized by a long terminal elimination half-life (20 to 28 days) and a large between-patient variability in the pharmacokinetic profile in different subpopulations (8–11). Although its pharmacokinetic properties have been studied extensively, its pharmacodynamic properties in humans are less well studied, with available pharmacodynamic information (e.g., 50% effective concentration [EC_50_]) being mostly extrapolated from *in vitro* data (5, 12, 13), which might not always represent the pharmacodynamic properties in humans. Pharmacodynamic models of piperaquine in patients have been published previously in Plasmodium vivax malaria (14) and in chemoprevention of seasonal malaria (15). Knowledge of its key pharmacodynamic parameters in humans (e.g., EC_50_) would provide information for improving current treatments using ACTs and assisting with dose selection in new antimalarial therapies (e.g., triple combinations).

The induced blood-stage malaria (IBSM) model has been extensively used to investigate the activity of antimalarial drugs in humans, including piperaquine (16). In the IBSM model, healthy volunteers are inoculated with P. falciparum-infected erythrocytes, which allows for an evaluation of the activity of antimalarial drugs against the asexual blood stages of the parasites. Moreover, the IBSM model allows the investigation of parasite dynamics both before and after the antimalarial drug treatment, which is not possible with data from field studies where only parasite elimination can be studied.

The aim of this study was to develop a pharmacokinetic-pharmacodynamic model describing the parasite dynamics in healthy volunteers inoculated with blood-stage P. falciparum parasites using the IBSM model. The pharmacokinetic-pharmacodynamic model that was developed was then used to predict treatment failures in the presence of multidrug-resistant infections and characterize the ideal partner drug for triple-combination therapy for these infections.

## RESULTS

### Population pharmacokinetic model of piperaquine.

**FIG 1:**
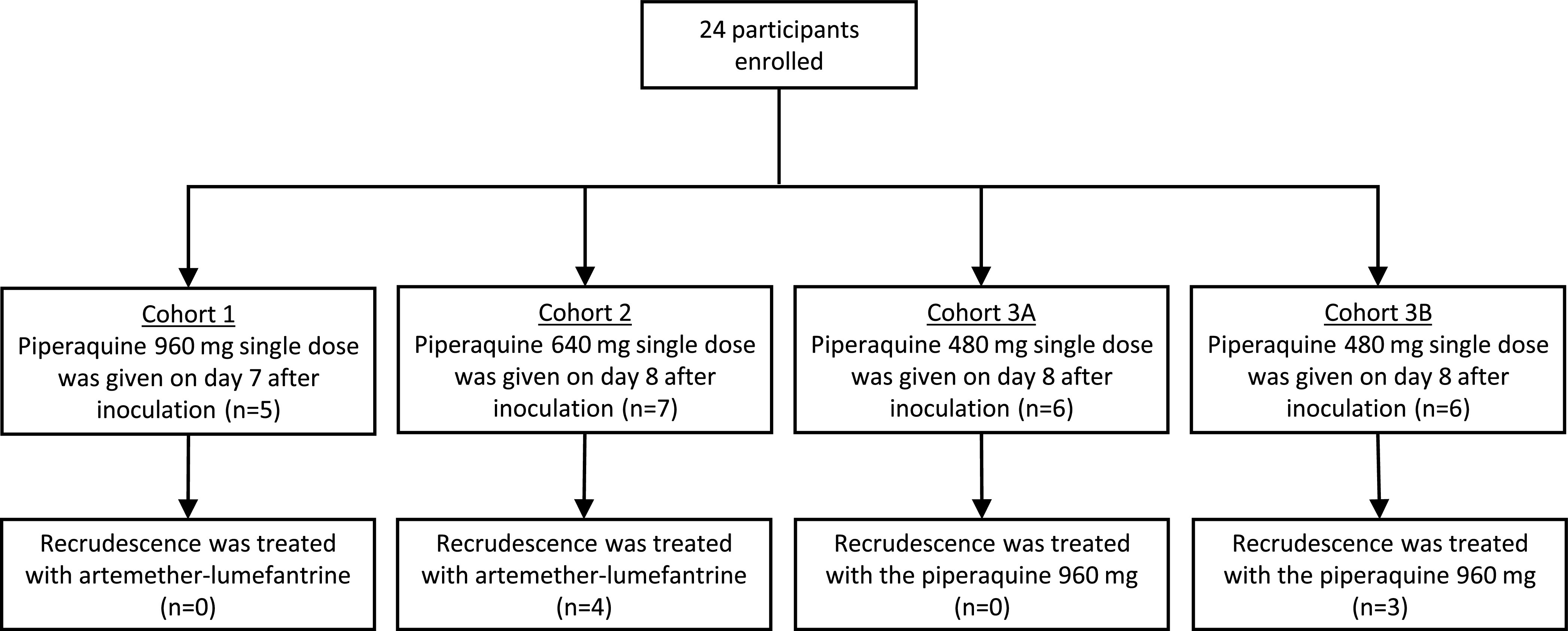
Cohort diagram.

A total of 475 piperaquine plasma concentrations were collected from 24 participants from 4 cohorts (Fig. 1). The characteristics of participants are shown in Table 1. Piperaquine concentrations were measured to be above the lower limit of quantification in all plasma samples. The pharmacokinetic properties of piperaquine were best described by a three-compartment disposition model, characterizing the triphasic disposition of the drug (difference in objective function value [ΔOFV] = –41.3; 2 degrees of freedom [df], compared with two-compartment disposition model). An additional disposition compartment for piperaquine did not improve the model fit (ΔOFV = 0). The absorption process was best described by two-transit compartments, resulting in a substantial improvement compared with a traditional first-order absorption model (ΔOFV = –230; 0 df). Interoccasion variability (IOV) was evaluated on absorption parameters, i.e., relative bioavailability (F) and mean absorption transit time (MTT), which improved the model fit significantly (ΔOFV = –37.8; 2 df). An additive error model described the data accurately, and a combined additive and proportional error model did not improve the model fit (ΔOFV = 0.05). Implementation of body weight, using an allometric function, improved the model (ΔOFV = –4.44; 0 df). No additional significant covariates were found during the stepwise covariate search. The model evaluations indicated satisfactory results, with no obvious trends in the goodness of fit plots (see Fig. S1A to D in the supplemental material). Similarly, the visual predictive check (Fig. S1E) demonstrated a good predictive performance of the model. The numerical predictive check (*n *= 2,000) showed that 3.2% (95% confidence interval [CI], 1.5% to 10.1%) of piperaquine observations were below the simulated 90% prediction interval and 5.1% (95% CI, 1.3% to 9.9%) were above. The bootstrap results demonstrated acceptable robustness of the piperaquine population pharmacokinetic model. The final pharmacokinetic parameter estimates of piperaquine, with precision and shrinkage are summarized in Table 2.

**TABLE 1:** Participant characteristics

Characteristic	Values
Age (yrs)^*a*^	22.5 (18–32)
BMI^*b*^ (kg/m^2^)^*a*^	22.8 (18.3–27.9)
Height (cm)^*a*^	173 (149–186)
Weight (kg)^*a*^	69.3 (51.1–86.9)
Sex^*c*^	
Male	15 (62.5)
Female	9 (37.5)
Race^*c*^	
Australian White	20 (83.3)
Australian Asian	1 (4.17)
Other	3 (12.5)

a Values are median (range).

b BMI, body mass index.

c Values are *n* (%).

**TABLE 2:** Population pharmacokinetic parameter estimates from the final pharmacokinetic model of piperaquine

Parameter^*a*^	Population estimate^*b*^ (% RSE)^*c*^	Population estimate^*b*^ 95% CI^*c*^	IIV ^*b*^ or IOV^*b*^ (% RSE)^*c*^	IIV ^*b*^ or IOV^*b*^ 95% CI^*c*^	Shrinkage (%)
F	1 Fixed		43.7 (14.4), 19.0 (28.8)^*d*^	28.1–56.0, 5.98–29.1^*d*^	6.22, 64.2^*d*^
MTT (h)	3.05 (5.44)	2.66–3.31	39.4 (29.7)^*d*^	12.3–64.5^*d*^	45.0^*d*^
CL/F (liter/h)	52.4 (10.4)	42.2–63.4	39.2 (17.2)	22.6–50.8	15.3
V_C_/F (liter)	542 (22.1)	349–842			
Q_1_/F (liter/h)	2,400 (41.1)	1,210–4,670	298 (18.7)	121–695	12.1
V_P1_/F (liter)	3,320 (12.7)	2,580–4,220	27.4 (34.0)	1.74–45.2	29.4
Q_2_/F (liter/h)	152 (12.3)	122–196	20.7 (43.5)	0.561–38.1	53.3
V_P2_/F (liter)	13,500 (13.0)	10,500–17,300	18.8 (40.1)	0.412–30.4	51.4
σ	0.114 (5.04)	0.091–0.134			10.0

a F, relative bioavailability; MTT, mean transit time; CL/F, apparent oral clearance; V_C_/F, apparent central volume of distribution; Q/F, apparent intercompartmental clearance from central compartment to peripheral compartment; V_P_/F, apparent peripheral volume of distribution; and σ, residual unexplained variability.

b Population mean parameters estimated from NONMEM, based on a typical individual weighing 69.3 kg. Interindividual variability (IIV) and interoccasion variability (IOV) are presented as the coefficient of variation (% CV), calculated as $100\times \sqrt{\text{exp}(\text{estimate})-1}$.

c Based on nonparametric bootstrap diagnostics (*n *= 1,000). Parameter precision is presented as relative standard deviation (% RSE), calculated as 100 × standard deviation/mean value.

d Values for interoccasion variability.

### P. falciparum parasite growth model.

A recently published pooled analysis of parasite growth data from malaria volunteer infection studies, using a larger data set including results from this study, reported a parasite life cycle of 38.8 h (17). Thus, the parasite life cycle was fixed to 38.8 h in all growth models evaluated in the current study. A total of 8.82% of the observed parasite density values were below the lower limit of detection (LOD), and these data were successfully handled using the M3 method (15). Three parasite growth models were evaluated, as described below.

### Log-linear growth model and sine-wave growth model.

The parasite growth rate (k_G_) estimated from a log-linear growth model was 0.066 h^−1^, equivalent to a parasite multiplication rate per life cycle (PMR_LC_; parasite multiplication rate, equivalent to the number of new merozoites released from 1 schizont) of 12.9-fold (95% CI, 11.5 to 14.3). Adding interindividual variability on parasite life cycle improved the model fit (ΔOFV = –6.29; 1 df). Additional interindividual variability on k_G_ did not improve the model fit further (ΔOFV = 0.002; 1 df). For the sine-wave growth model, k_G_ was estimated at 0.065 h^−1^, equivalent to PMR_LC_ of 12.6-fold (95% CI, 11.1 to 14.0). The sine-wave growth model improved the model fit compared with the log-linear model (ΔOFV = −69.7; difference in Bayesian information criterion [ΔBIC] = −59.5). Adding interindividual variability on k_G_ improved the model fit (ΔOFV = –20.0; 1 df). Additional interindividual variability on parasite life cycle did not improve the model fit further (ΔOFV = 0.03; 1 df). The final parameter estimates of the log-linear growth model and the sine-wave growth model are summarized in Table S1 and S2, respectively, in the supplemental material. The goodness of fit of the growth phase data using log-linear and sine-wave growth models showed an acceptable overall goodness of fit with no obvious systematic model misspecifications (see Fig. S2 in the supplemental material).

### Semimechanistic growth model.

A semimechanistic growth model mimicking the natural P. falciparum malaria parasite life cycle was developed. The rate constant of parasite maturation (k_MAT_) and the rate constant of schizont rupture (k_RUP_) were fixed to an arbitrary high value of 2 in order to ensure that all parasites matured from being young rings to mature parasites and that schizont rupture took place at 38.8 h (resulting in parasite multiplication). The fraction of parasite sequestration (F_SQ_) was not identifiable with the data available. A sensitivity analysis of F_SQ_ was performed by fixing this parameter, ranging from 40% to 90% with a 10% increase each time. No alteration was observed in the OFV or in model goodness-of-fit diagnostics, suggesting identifiability issues of F_SQ_. However, the observed growth phase data showed a recurring 10-fold drop in total circulating parasites (P_CIR_), suggesting that the fraction of parasite sequestration should be approximately 90%. Thus, the F_SQ_ was fixed to 90% according to these observations. The onset of sequestration (T_SQ_) was estimated at 29.1 h (95% CI, 27.7 to 29.7 h). Parasite growth rate was estimated as 0.0710 h^−1^ (95% CI, 0.0682 to 0.0771 h^−1^), equivalent to a PMR_LC_ of 15.7-fold (95% CI, 14.1 to 19.9). Adding interindividual variability on the parasite life cycle and PMR_LC_ improved the model fit (ΔOFV = –48.6; 2 df).

The developed semimechanistic growth model described the observed data well and the goodness of fit of the semimechanistic growth model demonstrated a better model fit than the log-linear growth model (ΔOFV = −33.2, ΔBIC = −22.9) and also in terms of describing the net decrease in total circulating parasite number, a consequence of parasite sequestration (Fig. S2). The semimechanistic growth model did not result in a better model fit, in terms of ΔOFV and ΔBIC, compared with the sine-wave growth model (ΔOFV = 36.5, ΔBIC = 36.5), but demonstrated a similar goodness of fit. However, the semimechanistic growth model gave some advantages by describing the maturation of parasites, sequestration of mature parasites, synchronicity of infections (i.e., synchronous parasite maturation and multiplication resulting in periodic bursts of red blood cells and the release of young parasites), and multiplication of parasites, as described in natural infections with P. falciparum. These advantages provided additional flexibility for investigating drug effects on specific stages of the parasite life cycle. Thus, the semimechanistic growth model was carried forward for further investigation. The final parameter estimates with precision and shrinkage of model parameters are presented in Table 3.

**TABLE 3:** Population pharmacodynamic model parameter estimates

Parameter^*a*^	Population estimate^*b*^ (% RSE)^*c*^	Population estimate^*b*^ 95% CI^*c*^	IIV^*b*^ (% RSE)^*c*^	IIV^*b*^ 95% CI^*c*^	Shrinkage (%)
Semimechanistic growth model					
P1 (h)	0–9.7 Fixed				
P2 (h)	9.7–T_PC_ Fixed				
P3 (h)	T_SQ_–T_PC_ Fixed				
F_SUR_ (%)	5 Fixed				
T_PC_ (h)	38.8 Fixed		6.00 (24.8)	5.11–8.50	34.5
k_MAT_ (h^−1^)	2 Fixed				
T_SQ_ (h)	29.1 (4.84)	27.7–29.7			
F_SQ_ (%)	90 Fixed				
k_RUP_ (h^−1^)	2 Fixed				
PMR_LC_	15.7 (8.43)	14.1–19.9	18.3 (27.7)	15.4–33.3	14.6
*In vivo* parasiticidal effect of piperaquine					
*E*_max_ (h^−1^)	0.289 (5.31)	0.262–0.321	23.6 (26.0)	5.20–30.0	18.7
EC_50_ (ng/ml)	5.43 (29.4)	1.77–7.33	114 (38.6)	67.0–760	30.6
γ	2.8 Fixed				
σ	4.69 (4.80)	3.81–5.55			7.69

a P1, age of circulating small rings; P2, age of circulating large rings, trophozoites and schizonts; P3, age of sequestered trophozoites and schizonts; F_SUR_, fraction of parasite survival after inoculation; T_PC,_ duration of parasite life cycle; k_MAT_, first-order rate constant for parasite maturation; T_SQ_, onset of parasite sequestration; F_SQ_, fraction of parasites sequestration; k_RUP_, first-order rate constant of schizont rupture; PMR_LC_, parasite multiplication rate given as fold increase per life cycle; *E*_max_, maximum parasite killing rate of piperaquine; C_P_, piperaquine plasma concentration; EC_50_, plasma concentration of piperaquine associated with half of maximum parasite killing rate; γ, hill factor; and σ, residual unexplained variability.

b Population mean parameters estimated from NONMEM, based on a typical individual weighting 69.3 kg. Interindividual variability (IIV) and interoccasion variability (IOV) are presented as the coefficient of variation (% CV), calculated as $100\times \sqrt{\text{exp}(\text{estimate})-1}$.

c Based on nonparametric bootstrap diagnostics (*n *= 1,000). Parameter precision is presented as relative standard deviation (% RSE), calculated as 100 × standard deviation/mean value.

### *In vivo* parasiticidal effect of piperaquine.

**FIG 2:**
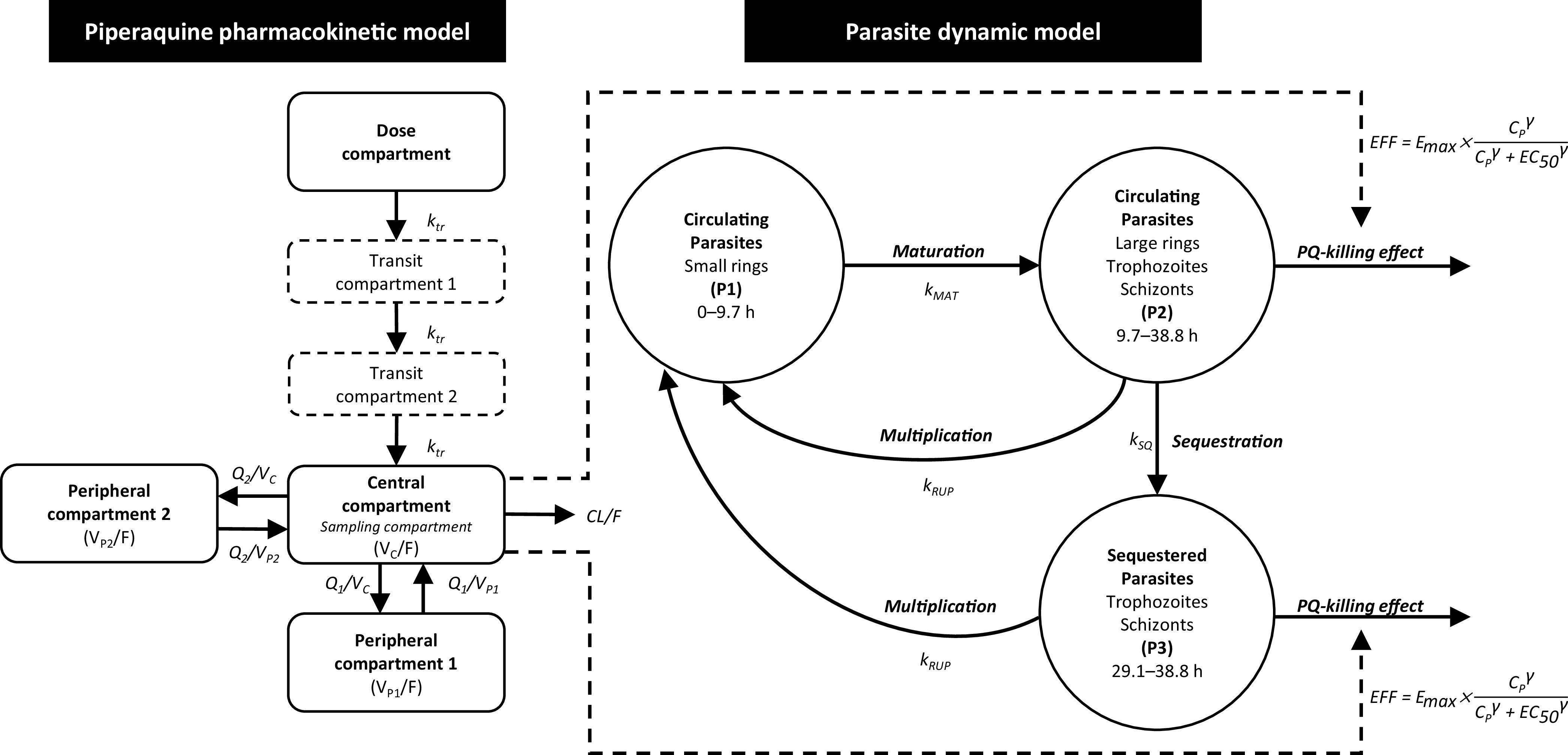
The schematic of final pharmacokinetic-pharmacodynamic model describing the semimechanistic model of P. falciparum malaria parasites and the final pharmacokinetic model of piperaquine. In the piperaquine pharmacokinetic model (left), F represents relative bioavailability, k_tr_ represents transit rate constant, CL/F represents apparent oral clearance, V_C_/F represents apparent central volume of distribution (PK sampling compartment), Q/F represents intercompartmental clearance from central compartment to peripheral compartment, and V_P_/F represents apparent peripheral volume of distribution. In the parasite dynamic model (right), circulating parasites (P1 + P2) represent the observed parasitemia, k_MAT_ represents first-order rate constant of parasite maturation, k_SQ_ represents first-order rate constant of parasite sequestration, and k_RUP_ represents first-order rate constant of schizont rupture. The killing effect of piperaquine (EFF) was described by an *E*_max_ function; *E*_max_ represents the maximum parasite killing rate of piperaquine, C_P_ represents piperaquine plasma concentration, and EC_50_ represents plasma concentration of piperaquine associated with half of maximum parasite killing rate.

**FIG 3:**
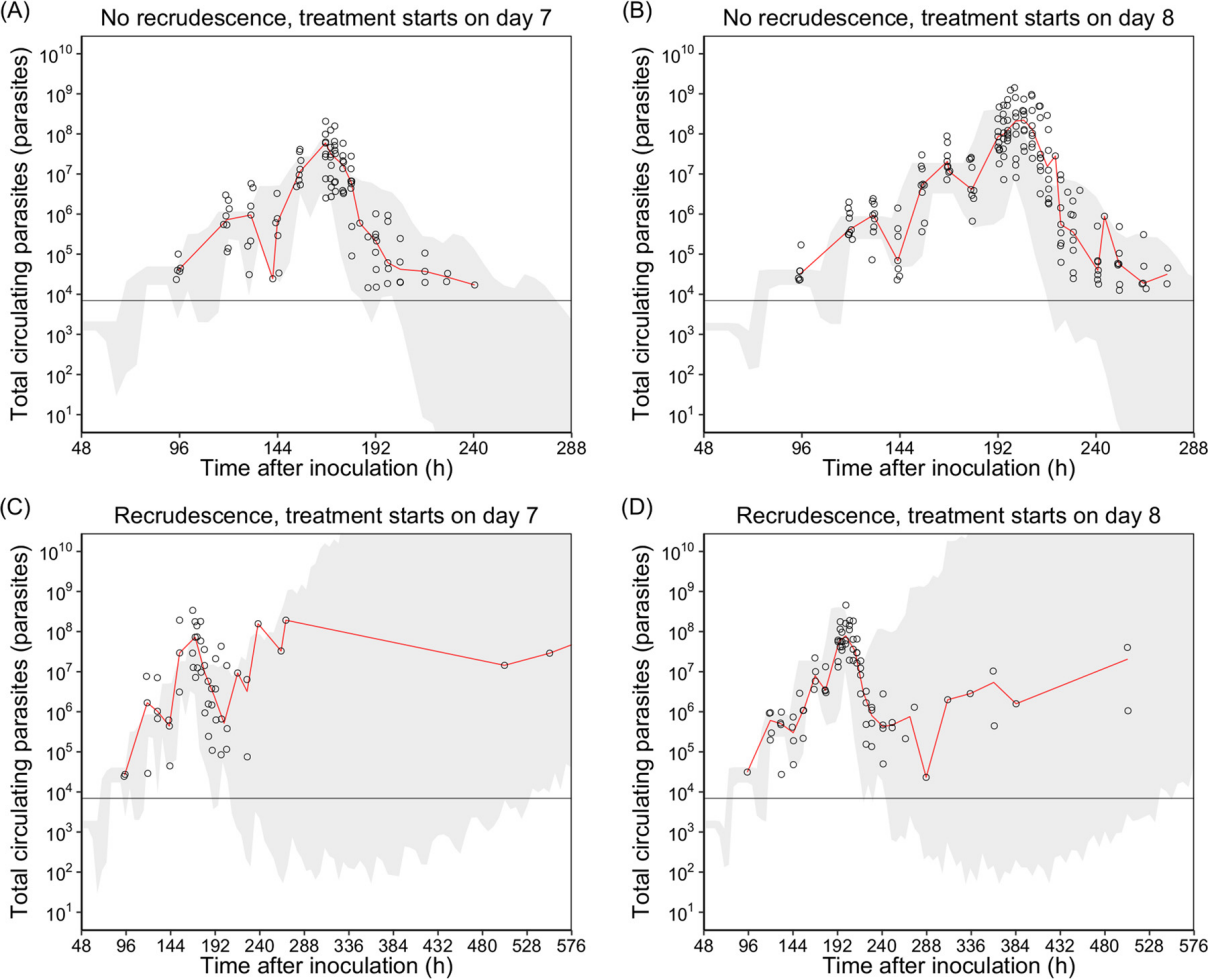
The simulated 90% prediction interval from the final pharmacokinetic-pharmacodynamic model (*n *= 1,000). The open circles represent the observed total circulating parasites. Solid red lines represent the 50th percentiles of the observations, and horizontal black lines represent the lower limit of parasite detection (LOD). The shaded areas represent the 90% prediction intervals of the simulation.

The pharmacokinetic-pharmacodynamic model was developed to investigate the *in vivo* parasiticidal effect of piperaquine. The schematic of the final pharmacokinetic-pharmacodynamic model is shown in Fig. 2. The parasiticidal effect of piperaquine (EFF) was added as a direct effect (equations 14 and 15 in Materials and Methods) to the mature parasite compartments (P2 and P3), describing the drug-dependent parasite elimination adequately. The model estimated the maximum parasite killing rate of piperaquine (*E*_max_) as 0.289 h^−1^ (95% CI, 0.262 to 0.323 h^−1^) with an estimated EC_50_ of 5.43 ng/ml (95% CI, 1.68 to 7.33 ng/ml), resulting in a median parasite reduction ratio per life cycle (PRR_LC_) of 2.68 × 10^2^, 2.89 × 10^2^, and 3.02 × 10^2^ when piperaquine was given as a single dose of 480, 640, and 960 mg piperaquine phosphate, respectively. The median minimum inhibitory concentration (MIC) of piperaquine derived from the 24 healthy volunteers was 2.87 ng/ml (95% CI, 1.87 to 18.3 ng/ml). The goodness-of-fit diagnostics of the final pharmacokinetic-pharmacodynamic model are presented in Fig. S3 in the supplemental material, simulation-base diagnostics (i.e., visual predictive checks) are presented in Fig. 3, and individual plots of the final pharmacokinetic-pharmacodynamic model are presented in Fig. S4 in the supplemental material. Parameter estimates from the final pharmacokinetic-pharmacodynamic model are presented in Table 3.

### Simulations of clinical scenarios.

In order to simulate treatment scenarios of drug-resistant infections, dihydroartemisinin was used as a representative artemisinin derivative. The parasiticidal effect of dihydroartemisinin was adjusted based on the observed parasite clearance half-life from the Tracking Resistance to Artemisinin Collaboration (TRAC) study data (18). The adjusted dihydroartemisinin *E*_max_ values were 0.477 h^−1^ and 0.216 h^−1^ for sensitive and resistant infections, respectively (see Fig. S5 in the supplemental material).

Various degrees of piperaquine resistance were represented by doubling (10.9 ng/ml), tripling (16.3 ng/ml), and quadrupling (21.7 ng/ml) the estimated EC_50_ of sensitive parasites (5.43 ng/ml). In a symptomatic infection (initial total circulating parasites of 10^10^), these simulations predicted a similar probability of treatment failure in piperaquine resistance alone compared with dihydroartemisinin resistance alone (2.58% versus 1.81%). The probability of treatment failure increased with multidrug-resistant infections, resulting in 23.6% treatment failures in the presence of dihydroartemisinin resistance and a high-degree of piperaquine resistance (EC_50_ of 21.7 ng/ml). A similar trend of treatment failure was predicted in asymptomatic infections (initial total circulating parasites of 10^6^). A summary of the treatment failure probability of each drug-resistant scenario is presented in Table 4. Additionally, simulations to inform on suitable pharmacokinetic-pharmacodynamic characteristics for candidate partner drugs used in triple-combination therapy demonstrated that the addition of a hypothetical drug with a PRR_LC_ of ≥10^2^ and total therapeutic duration of ≥2 weeks had a >99% probability of successful treatment. The summary of treatment failure probability with simulated hypothetical partner drug activities against multidrug-resistant infections is presented in Table 5.

**TABLE 4:** Predicted probability of treatment failure associated with different levels of drug resistance

Drug-resistant scenario^*a*^	Probability of treatment failure (%)	
	Asymptomatic infection^*b*^	Symptomatic infection^*c*^
DHA sensitive (*E*_max_ = 0.477), PQ sensitive (EC_50_ = 5.4 ng/ml)	<1.00	<1.00
DHA resistant (*E*_max_ = 0.216), PQ sensitive (EC_50_ = 5.4 ng/ml)	<1.00	1.81
DHA sensitive (*E*_max_ = 0.477), PQ resistant (EC_50_ = 10.9 ng/ml)	<1.00	2.58
DHA resistant (*E*_max_ = 0.216), PQ resistant (EC_50_ = 10.9 ng/ml)	2.44	8.06
DHA resistant (*E*_max_ = 0.216), PQ resistant (EC_50_ = 16.3 ng/ml)	7.10	15.2
DHA resistant (*E*_max_ = 0.216), PQ resistant (EC_50_ = 21.7 ng/ml)	10.6	23.6

a DHA, dihydroartemisinin; PQ, piperaquine; *E*_max_, maximum parasite killing rate of dihydroartemisinin; and EC_50_, concentration of piperaquine associated with half of maximum parasite killing rate.

b Initial total circulating parasites for asymptomatic infection of 10^6^ parasites.

c Initial total circulating parasites for symptomatic infection of 10^10^ parasites.

**TABLE 5:** Predicted probability of treatment failure associated with treating a symptomatic infection with hypothetical triple combination therapy^*a*^

Drug-resistant scenario	Probability of treatment failure (%)						
	DHA + PQ^*b*^	Hypothetical drug characteristics					
		PRR_LC_	Duration of action (wks)				
			1	2	3	4	5
DHA resistant (*E*_max_ = 0.216), PQ resistant (2 × EC_50_ = 10.9 ng/ml)	8.1	10^1^	4.19	2.79	2.27	2.29	2.23
		10^2^	1.42	<1.00	<1.00	<1.00	<1.00
		10^3^	<1.00	<1.00	<1.00	<1.00	<1.00
DHA resistant (*E*_max_ = 0.216), PQ resistant (3 × EC_50_ = 16.3 ng/ml)	15.2	10^1^	9.35	5.60	5.31	5.46	5.46
		10^2^	3.65	<1.00	<1.00	<1.00	<1.00
		10^3^	<1.00	<1.00	<1.00	<1.00	<1.00
DHA resistant (*E*_max_ = 0.216), PQ resistant (4 × EC_50_ = 21.7 ng/ml)	23.6	10^1^	15.3	10.7	10.0	9.96	9.94
		10^2^	6.02	<1.00	<1.00	<1.00	<1.00
		10^3^	1.25	<1.00	<1.00	<1.00	<1.00

a The hypothetical drug was added to the standard 3-day dose of DHA-PQ (120/960 mg). DHA, dihydroartemisinin; PQ, piperaquine; *E*_max_, maximum parasite killing rate of dihydroartemisinin; EC_50_, concentration of piperaquine associated with half of maximum parasite killing rate; and PRR_LC_, parasite reduction ratio per parasite life cycle (38.8 h).

b Initial total parasite biomass for symptomatic infection of 10^10^ parasites.

## DISCUSSION

In the present study, a semimechanistic growth model describing P. falciparum parasite dynamics was successfully developed. The integration of piperaquine pharmacokinetics and parasite dynamics using data from a study conducted using the IBSM model allowed the estimation of the *in vivo* pharmacodynamic parameters of piperaquine.

The PMR_LC_ estimated from the log-linear growth model (12.9; 95% CI, 11.5 to 14.3), the sine-wave growth model (12.6; 95% CI, 11.1 to 14.0), and the semimechanistic growth model (15.7; 95% CI, 14.1 to 19.9) were all similar to those reported in a pooled analysis of growth data (16.4; 95% CI, 15.1 to 17.8) (17) and were also similar to the approximately 10-fold multiplication rate reported in natural infections (19, 20). During parasite growth model development, the log-linear and cosine-wave models estimated F_SUR_ as less than 1% with high relative standard errors of 57% and 41%, respectively. The semimechanistic growth model estimated F_SUR_ as 5.4% with a relative standard error of 19%. Pooling data from several studies conducted at QIMR Berghofer Medical Research Institute (QIMR), a separate pharmacokinetic/pharmacodynamic model estimated the fraction of survival for inoculated parasites as 5%. In order to avoid bias when comparing parasite growth models and the difficulties in estimating this parameter from the available data, F_SUR_ was fixed to 5% based on our semimechanistic growth model and the unpublished model from QIMR.

In the semimechanistic growth model, the mean starting time point of parasite sequestration was estimated at 29.1 h. Sequestration is not likely to start at the same time in all individuals, but the estimated interindividual variability in this parameter was large with low precision. This result is most likely due to the limited number of measurements of parasitemia across the parasite life cycle. Thus, interindividual variability was retained only in the length of the parasite life cycle and parasite multiplication rate in the final semimechanistic growth model.

The parasiticidal effect of piperaquine on P. falciparum predominantly affects late-stage parasites (trophozoites and schizonts) (18, 21, 22). Therefore, the parasiticidal effect of piperaquine was assumed to be against only the late-stage parasites in the semimechanistic growth model, both in peripheral circulation and in the sequestered vascular compartment. The goodness of fits and individual fits demonstrated that the developed model described the observed data adequately both in recrudescent and nonrecrudescent individuals. Simulation-based diagnostics of the final pharmacokinetic-pharmacodynamic model demonstrated a good predictive performance of cured individuals, but the simulated prediction intervals of individuals with recrudesce showed relatively large variability. This finding could be explained by the small proportion of recrudescent data available in this study.

The estimated *in vivo* EC_50_ of 5.43 ng/ml (95% CI, 1.68 to 7.33 ng/ml) was similar to the mean IC_50_ values of piperaquine against the 3D7 parasite strain, which was reported in two *in vitro* susceptibility studies (1.69 to 7.34 ng/ml) (23, 24). However, this estimated *in vivo* EC_50_ was lower than the *in vitro* IC_50_ value reported for sensitive infections in the field. The *in vitro* IC_50_ of piperaquine reported in 2011 in three sites in Cambodia were 10.7 ng/ml (interquartile range [IQR], 7.34 to 15.5 ng/ml) in Ratanakiri, 10.3 ng/ml (IQR, 8.09 to 14.0 ng/ml) in Preah Vihear, and 10.5 ng/ml (IQR, 6.37 to 18.2 ng/ml) in Pursat (5). The lower estimated EC_50_ in this study than IC_50_ values reported in the field might be partially explained by the different genetic background of the parasites, i.e., the 3D7 strain used in the current study was originally isolated from Africa and is sensitive to chloroquine and several antimalarial drugs, including piperaquine (23–25). The median PRR_LC_ derived from the final pharmacodynamic model was 2.68 × 10^2^ to 3.02 × 10^2^ at standard therapeutic doses. These values are similar to the 48-h parasite reduction ratio (PRR_48_) of 10^2^ to 10^5^ reported in the literature (26, 27), which correspond to a PRR_LC_ of approximately 4.14 × 10^1^ to 1.10 × 10^4^ when corrected for a shorter life cycle length. However, the reduction ratio reported in the present study is in the lower end of what has been reported in the literature. This difference can be explained by several factors such as the synchronicity of the infection, potential differences between the laboratory strain used in this model and clinical isolates (e.g., parasite life cycle, parasite multiplication rate, and parasite susceptibility to piperaquine), and the difference in background immunity in malaria-naive volunteers versus patients from regions of malaria endemicity with pre-exposure to malaria.

Simulations of multidrug-resistant scenarios predicted a probability of treatment failure that was somewhat lower than that reported in the clinical settings in Cambodia, especially in the scenario with an extremely resistant infection (i.e., 4-fold increase in EC_50_ as reported in Pursat). The rates of clinical recrudescence reported from this study in 2013 were 2%, 16%, and 46% in Ratanakiri, Preah Vihear, and Pursat, respectively. The *in vitro* IC_50_ of piperaquine in these 3 study sites was increased by approximately 2-, 3-, and 4-fold, respectively, in 2013 compared with the values reported in 2011 (4, 5). Assuming the same increase in EC_50_ in the present study resulted in simulated failure rates of 8.1%, 15.2%, and 23.6%. The difference in the predicted proportion of treatment failures versus observed clinical failure rates could possibly be a result of a difference in parasite dynamics as discussed above and/or the pharmacokinetic properties of piperaquine, which might differ between healthy volunteers and patients. Differences in treatment adherence could also partly explain the difference. Moreover, the assumption of the model with respect to the dihydroartemisinin parasite killing effect (*E*_max_), based on the parasite clearance half-life values associated with artesunate in sensitive and resistant infections from a previous study (28), might not be a perfect representation of dihydroartemisinin resistance occurring in the field. The simulations in the current study enabled a prediction of the probability of treatment failure when an additional partner drug was added to the conventional dihydroartemisinin-piperaquine regimen. This information could help with the selection of new combination therapies and optimization of dosing regimens. In all simulations of treatment failures, an additive drug effect was assumed for drugs included in the treatment. We believe this is the most conservative approach, considering the limited information available on drug synergisms/antagonisms of antimalarial drugs. However, applying a different drug interaction could yield different results. A previous study (29) showed that a model incorporating a different magnitude of interactions between antimalarial drugs, using dihydroartemisinin-piperaquine plus mefloquine as an example, predicted a reduced probability of treatment cure when the level of antagonism between piperaquine and mefloquine was high.

Some limitations of the semimechanistic model developed in the current study include characterization of the fraction of sequestered parasite (F_SQ_) and the relatively high uncertainty in estimation of the time to sequestration. These issues could be overcome by more frequent measurements of parasite densities during the growth phase. Furthermore, stage-specific parasite measurements could also enhance the robustness and reliability of the model. The semimechanistic growth model resulted in improved model fit compared with the log-linear model but did not result in a better model fits, in terms of ΔOFV and ΔBIC, compared with the sine-wave growth model. Nevertheless, we believe that a semimechanistic growth model, based on observed biological processes in the parasite life cycle, is preferable to an empirical description of the data and a more useful tool for translational simulations, especially since the developed semimechanistic model can be modified to include antimalarial drugs with different mechanisms of action (i.e., drug effect can be incorporated at different stages in the parasite life cycle depending on the mechanism of action). Another limitation is the potential differences between the P. falciparum strain used in the IBSM model (3D7) and those in patients with clinical malaria. If the P. falciparum strain used in the IBSM model is more drug susceptible than the strains in clinical infections, this would lead to an overestimation of the drug-mediated killing of parasites. However, the pharmacokinetic-pharmacodynamic model structure, incorporating a semimechanistic growth model, allows the flexibility to evaluate drug effects to a specific stage of the parasite life cycle. Furthermore, the model explained the processes described in natural P. falciparum malaria infections, including maturation of parasites, sequestration of mature parasites, synchronicity of infections, and multiplication of parasites. The implementation of this model structure to growth phase data from a large pool of malaria volunteer infection studies could further confirm the robustness of the model and hopefully allow for all model parameters to be estimated.

In conclusion, an *in vivo* semimechanistic model of parasite growth and clearance was developed in participants inoculated with P. falciparum malaria, and model parameters (*E*_max_, EC_50_, and PRR_LC_) associated with piperaquine pharmacokinetic-pharmacodynamic effects were estimated. This semimechanistic parasite model provides important insights and could be an important tool in the development of novel triple-combination therapies and for dose optimization of piperaquine and other antimalarial drugs.

## MATERIALS AND METHODS

### Study design.

Pharmacokinetic and pharmacodynamic data were collected from 24 healthy volunteers who participated in a previously described phase-Ib single-center clinical trial (26). In brief, the study was conducted at the contract research organization Q-Pharm (Brisbane, Australia). Malaria-naive subjects aged 18 to 50 years old who met all of the inclusion and none of the exclusion criteria were eligible to enroll in the study. The study protocol was approved by the QIMR Berghofer Human Research Ethics Committee and was registered in the Australian and New Zealand Clinical Trials Registry (ANZCTRN12613000565741). Eligible participants were intravenously inoculated with approximately 1,800 viable P. falciparum-infected human erythrocytes (chloroquine-susceptible 3D7 strain) on day 0. A single dose of piperaquine was given to participants in four different cohorts. The doses of piperaquine phosphate were 960 mg (cohort 1), 640 mg (cohort 2), and 480 mg (cohorts 3a and 3b). Piperaquine was administered on day 7 (cohort 1 and 3b; *n* = 11) or day 8 (cohort 2 and 3a; *n* = 13) after inoculation. The parasite density in inoculated participants was quantified using a quantitative PCR (qPCR) that targets the P. falciparum 18S rRNA gene (30). Treatment for malaria recrudescence consisted of artemether-lumefantrine in cohorts 1 and 2 and a second dose of piperaquine (960 mg) in cohorts 3a and 3b. At the end of the study, artemether-lumefantrine was given to all participants as a curative malaria treatment. Plasma concentrations of piperaquine were measured using liquid chromatography and mass spectrometry. The plasma samples were collected before piperaquine administration; at 0.5, 1, 2, 3, 4, 6, 8, 12, 24, 48, 72, 96, and 144 hours after piperaquine administration; at 8, 11, and 14 days after treatment; and at the end of study (day 28 for cohorts 1 and 2, day 37 for cohort 3a, and day 35 for cohort 3b). For cohorts 3a and 3b, an additional sample was taken 18 days after piperaquine administration. The range of the assay was 0.5 to 1000 μg/liter for piperaquine. The coefficient of variation across four different concentration levels was 1.2% to 4.4% (*n* = 10) for the intraassay and 2.5% to 6.6% (*n* = 10) for the interassay comparisons. The accuracy of the assay was 97% to 104% (*n* = 10).

### Pharmacometric analysis.

The population pharmacokinetic and pharmacodynamic analysis was performed using nonlinear mixed-effects modeling in NONMEM, version 7.4 (Icon Development Solution, Ellicott City, MD). RStudio version 1.2.1335 (31), Xpose version 4.0, Pirana version 2.9.4 (32), and Pearl-speaks-NONMEM (PsN) version 4.7.0 (33) were used for model diagnostics and visualization of results. Piperaquine base plasma concentrations were transformed into their natural logarithms prior to pharmacokinetic model development. None of the samples had piperaquine concentrations measured below the lower limit of quantification. The first-order conditional estimation method with interaction (FOCE-I) was used throughout the pharmacokinetic model building process. The average parasite density from qPCR measurements (parasites/ml) was transformed into the total circulating parasites (P_CIR_; parasites) prior to pharmacodynamic model development (equation 2). The calculated total circulating parasite densities were transformed into their natural logarithms prior to pharmacodynamic model development. qPCR measurements below LOD (1 parasite/ml) were treated as categorical data and were modeled simultaneously with the reported continuous parasite density data above LOD, using the M3 method (34). Pharmacodynamic parameters were estimated using the FOCE-I and the Laplacian method (35). The difference in objective function value (ΔOFV) was used as a statistical criterion for discrimination between nested models. The difference in Bayesian information criterion (ΔBIC) was used when comparing nonnested models (36).

The descriptive performance of the model was assessed by goodness-of-fit diagnostics, and the predictive performance of the model was evaluated by simulation-based diagnostics. Eta and epsilon shrinkages were used to evaluate the reliability of the individual estimates and the ability to detect model misspecification in the goodness-of-fit diagnostics (37). The predictive performance of the final model was illustrated by visual and numerical predictive checks (*n *= 2,000). The 5th, 50th, and 95th percentile of the observed concentrations was overlaid with the 95% CI of each simulated percentile to detect model bias. Model robustness and nonparametric CI were evaluated using a bootstrap methodology (*n *= 1,000).

### Population pharmacokinetic model of piperaquine.

Pharmacokinetic parameters were assumed to be log-normally distributed, and interindividual variability was therefore implemented with an exponential function. Interoccasion variability, also implemented with an exponential function, was investigated to reflect the random variability between dosing occasions. An additive error model and a combined additive and proportional error model, both on the logarithmic scale, were evaluated. To evaluate the effect of body size on the pharmacokinetic properties of piperaquine, body weight was implemented as an allometric function on all clearance and volume of distribution parameters (equation 1).

(1) $$\theta_{i}=\theta \times e^{\eta_{i,\theta }}\times {\left(\frac{\text{B}{\text{W}}_{\text{i}}}{\text{B}{\text{W}}_{\text{median}}}\right)}^{n}$$

where *θ_i_* denotes individual clearance or individual volume of distribution parameter, θ denotes the typical value (population mean) of clearance or volume parameters, BW_i_ denotes individual body weight, BW_median_ denotes median body weight of the participants, and *n* was set to be equal to 0.75 for clearance parameters and 1 for volume parameters. Additional covariate relationships including age, sex, and race were examined using a stepwise forward inclusion (*P*  < 0.05, ΔOFV = –3.84), followed by stepwise backward elimination (*P* > 0.001, ΔOFV= –10.83) procedure.

### P. falciparum parasite growth model.

The initial total number of circulating parasites (P_CIR_) was fixed to 1,800 parasites (equivalent to the approximate number of viable inoculated parasites) for all investigated parasite growth models, and the fraction of parasite survival (F_SUR_) after inoculation was fixed to 5%, based on results from a pharmacokinetic/pharmacodynamic model using pooled induced blood-stage malaria data at QIMR (unpublished work). The parasite life cycle was fixed to 38.8 h (17). The average parasite density from qPCR measurements (parasites/ml) at each time point was transformed to the P_CIR_ based on individual body weight multiplied by the average blood volume which was assume to be 80 ml/kg (equation 2) (38).

(2) $${\text{P}}_{\mathrm{CIR}}=\text{average parasite density (parasites/ml) × body weight (kg) × 80 (ml/kg)}$$

The calculated number of total circulating parasites was transformed into natural logarithms for parasite dynamic model development. Initially, only the growth-phase data were used to develop the parasite growth model (Fig. S6). Three different types of models were evaluated, namely, log-linear growth model, sine-wave growth model, and a semimechanistic growth model. The parasite dynamic model that best described the observed data was carried forward to evaluate parasite dynamics after piperaquine administration. The details of each parasite growth model are described below.

### Log-linear growth model and sine-wave growth model.

The log-linear growth model used to describe parasite growth data was implemented using a differential equation to explain the change of parasite density with time (equation 3).

(3) $$\frac{d{\text{P}}_{\mathrm{CIR}}}{dt}={\text{P}}_{\mathrm{CIR}}\times k_{G}$$

where *P_CIR_* denotes the number of total circulating parasites (parasites) and *k_G_* denotes parasite growth rate (h^−1^). The sine-wave model used in the previously published pooled analysis of parasite growth data from volunteer infection studies (17) was implemented to describe parasite growth data in the current study (equation 4).

(4) $$\text{ln}\left({\text{P}}_{\mathrm{CIR}}\right)=a+k_{G}\times \mathrm{time}+C\times \text{sin}\left[\left(\frac{2\times \pi }{T_{\mathrm{PC}}}\right)\times \mathrm{time}+k\right]$$

where, ln(*P_CIR_*) denotes the natural logarithm of total circulating parasites (parasites), *a* denotes y-intercept (i.e., the P_CIR_ at time zero), *time* denotes time after parasite inoculation (h), *C* denotes sine-wave amplitude, *T_PC_* denotes duration of the parasite life cycle (fixed to 38.8 h), and *k* denotes sine-wave phase shift. The overall parasite multiplication rate per life cycle (PMR_LC_) from the log-linear growth and sine-wave growth model was calculated using the estimated parasite growth rate and the duration of the parasite life cycle (equation 5).

(5) $$\mathrm{PM}{\text{R}}_{\mathrm{LC}}=e^{\left(T_{\mathrm{PC}}\times k_{G}\right)}$$

### Semimechanistic growth model.

**FIG 4:**
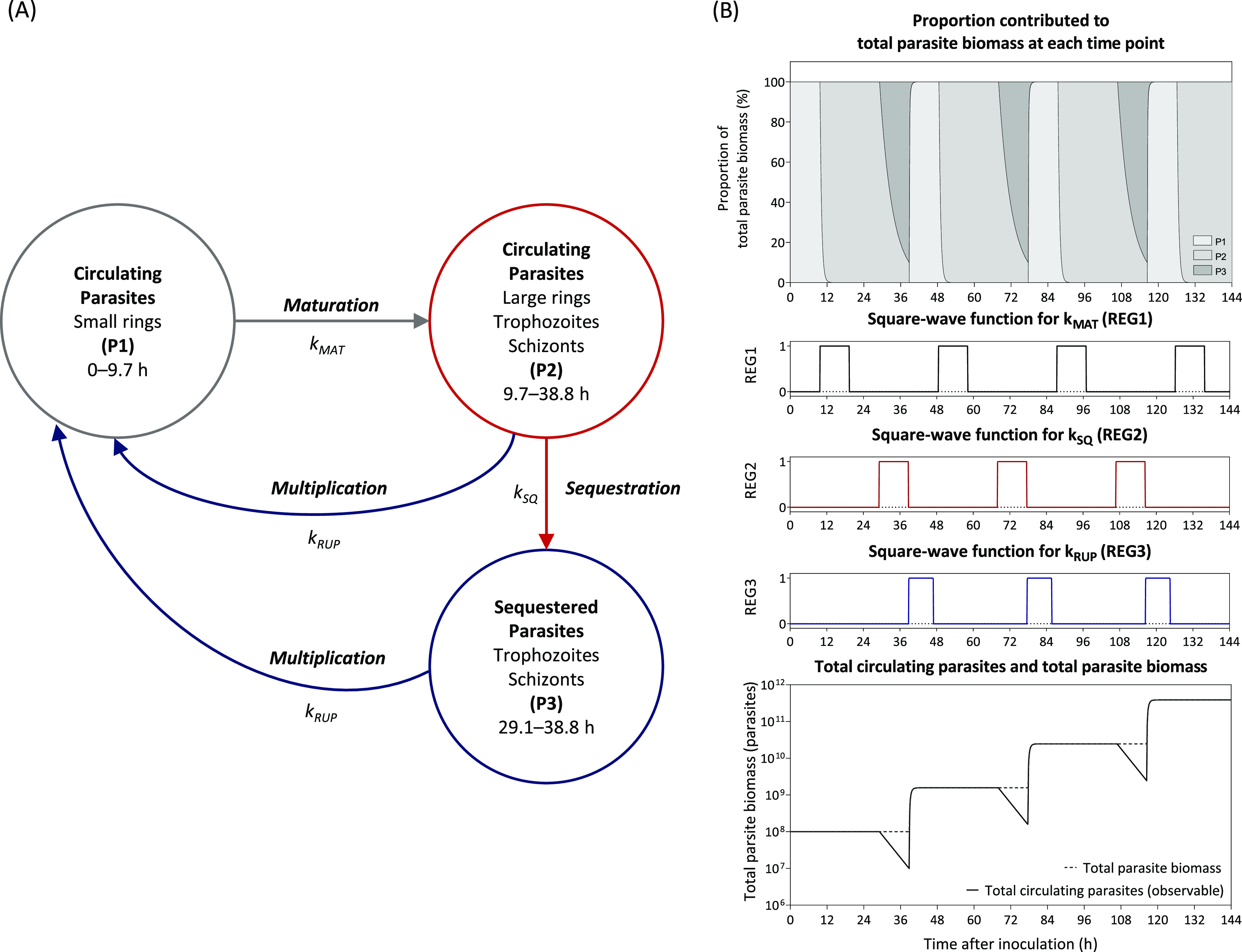
Semimechanistic growth model describing P. falciparum parasite dynamics. The left panel demonstrates the structure of the parasite growth model; (P1) represents small ring parasites that are circulating in the peripheral blood; (P2) represents the large rings, trophozoites, and schizonts that are circulating in the blood; (P3) represents the matured sequestered parasites; k_MAT_ represents first-order rate constant of parasite maturation [REG1 × 2(fixed)]; k_SQ_ represents first-order rate constant of parasite sequestration (REG2×k_SQ_); and k_RUP_ represents first-order rate constant of schizont rupture [REG3 × 2(fixed)]. The right panel demonstrated the sine-wave function used to regulate the parasite dynamics in each compartment and the associated parasite number at each stage of parasite life cycle. The equations used to generate the sine-wave function are presented in the supplemental material (NONMEM code).

The semimechanistic model was developed based on prior knowledge of the P. falciparum life cycle. Time windows corresponding to P. falciparum parasite stages were based on the microscopic observations previously reported (39). These time windows were corrected for a shorter life cycle length used in the current study (38.8 h). The proposed model consisted of three parasite compartments (Fig. 4). The first parasite compartment (P1) represents the small rings that are circulating in the peripheral blood. The second parasite compartment (P2) represents the large rings, trophozoites, and schizonts that are also circulating in the blood. These two parasite compartments represent the total circulating parasites in the peripheral blood, which can be measured by qPCR and microscopy. However, the total parasite biomass also includes sequestered parasites, which attach to endothelial cells. The third parasite compartment (P3) represents the sequestered parasites. Thus, combining the parasite number in all parasite compartments (P1, P2, and P3) yields a total parasite biomass in the body.

Three square-wave functions were used to regulate the movement of the parasites between each compartment at specific time periods (REG_1_, REG_2_, and REG_3_). These square-wave functions were set to change from 0 to 1 (i.e., “on-and-off”) during certain time periods to move parasites between the different parasite compartments. These square-wave functions were described by the following equations (equation 6 to 8).

(6) $$S_{1}=\text{sin}\left\{\frac{\left[\pi -\left(\frac{2\pi \times \text{S}{\text{H}}_{1}}{T_{\mathrm{PC}}}\right)\right]}{2}\right\}$$

(7) $$S_{2}=\text{sin}\left\{\left(\frac{2\pi \times \mathrm{time}}{T_{\mathrm{PC}}}\right)+\frac{\left[\pi -\left(\frac{2\pi \times \text{S}{\text{H}}_{1}}{T_{\mathrm{PC}}}\right)+\text{S}{\text{H}}_{2}\right]}{2}\right\}$$

(8) $$\mathrm{REG}=\sqrt{\frac{\left[{\left(S_{2}-S_{1}\right)}^{2}-\left(S_{2}-S_{1}\right)\right]}{\left[2\times \sqrt{{\left(S_{2}-S_{1}\right)}^{2}}\right]}}$$

where *S_1_* denotes the first sine-wave function, *S_2_* denotes the second sine-wave function, SH_1_ denotes time when the function remains in state 0 (h), SH_2_ denotes the peak shift time from state 0 to 1, *T_PC_* denotes duration of the parasite life cycle (fixed to 38.8 h), and *REG* denotes the square-wave function regulating parasite movement. SH_1_ and SH_2_ values used to generate each square-wave function (REG_1_ to REG_3_) for each parasite compartment were based on time windows corresponding to P. falciparum parasite stages (39). Details on how these functions were implemented in the model are shown in the supplemental material (NONMEM code).

In the semimechanistic growth model, the parasite number in P1 was initialized with 1,800 parasites according to the approximate number of viable inoculated parasites. The parasites in P1 mature over time and started moving to P2 at 9.7 to 19.4 h (REG_1_), and at 19.4 h, the entire parasite population in P1 moved to P2. The parasites in P2 continued to mature to schizonts, and either stayed in P2 or cytoadhered to the vascular endothelium and moved to P3 (sequestration). The sequestration of parasites was regulated (REG_2_) to occur during the latter half of the parasite life cycle at 19.4 to 38.8 h since sequestration begins at the large ring stage (40, 41). At the end of the parasite life cycle (38.8 h), the matured schizonts in P2 and P3 ruptured and released new merozoites back to compartment P1 (REG_3_). The PMR_LC_ associated with the rupture of schizonts was estimated. This semimechanistic growth model was described by the following differential equations system (equation 9 to 11).

(9) $$\frac{d\text{P}1}{\mathrm{dt}}=-\text{P}1\times k_{\mathrm{MAT}}\times \mathrm{RE}{\text{G}}_{1}+\left(\text{P}2+\text{P}3\right)\times k_{\mathrm{RUP}}\times \mathrm{RE}{\text{G}}_{3}\times \mathrm{PM}{\text{R}}_{\mathrm{LC}}$$

(10) $$\frac{d\text{P}2}{\mathrm{dt}}=\text{P}1\times k_{\mathrm{MAT}}\times \mathrm{RE}{\text{G}}_{1}-\text{P}2\times k_{\mathrm{SQ}}\times \mathrm{RE}{\text{G}}_{2}-\text{P}2\times k_{\mathrm{RUP}}\times \mathrm{RE}{\text{G}}_{3}$$

(11) $$\frac{d\text{P}3}{\mathrm{dt}}=\text{P}2\times k_{\mathrm{SQ}}\times \mathrm{RE}{\text{G}}_{2}-\text{P}3\times k_{\mathrm{RUP}}\times \mathrm{RE}{\text{G}}_{3}$$

where *k_MAT_* denotes the first-order rate constant for small rings to become mature parasites; *k_SQ_* denotes the first-order rate constant for parasite sequestration; *k_RUP_* denotes the first-order rate constant of schizont rupture; and *REG_1_*, *REG_2_*, and *REG_3_* denote the square-wave functions regulating the time and duration of parasite movement in each parasite compartment. Details of the square-wave functions used to regulate parasite movement are presented in the Fig. 4. In order to describe the parasite sequestration in a quantitative manner, the parasite sequestration rate was parameterized as a fraction of sequestered parasites (equation 12).

(12) $$k_{\mathrm{SQ}}=\frac{\text{ln}\left[\frac{100}{\left(100-F_{\mathrm{SQ}}\right)}\right]}{T_{\mathrm{PC}}-(T_{\mathrm{SQ}})}$$

where *F_SQ_* denotes the fraction of sequestered parasite (%) and *T_SQ_* denotes the onset of parasite sequestration (restricted to be between 19.4 and 38.8 h).

### *In vivo* parasiticidal effect of piperaquine.

The model that best described the parasite growth dynamics was carried forward and linked to the pharmacokinetic model of piperaquine. The final parameter estimates from the best performing model was used to impute individual growth time profiles. All total circulating parasite data, including the total circulating parasites after piperaquine administration, were used to estimate the parameters associated with the drug-dependent parasite elimination (Fig. S6). The parasiticidal effect of piperaquine was implemented as an *E*_max_ function (equation 13).

(13) $$\text{EFF}=\left(E_{\text{max}}\times \frac{C_{p}^{\gamma }}{C_{p}^{\gamma }+{\text{EC}}_{50}^{\gamma }}\right)$$

where EFF denotes the parasiticidal effect of piperaquine (h^−1^), *E*_max_ denotes the maximum parasite killing rate of piperaquine (h^−1^), *C_P_* denotes piperaquine plasma concentration (ng/ml), EC_50_ denotes the plasma concentration of piperaquine (ng/ml) associated with half of maximum parasite killing rate, and γ denotes the hill factor. Piperaquine was assumed to have an effect on the later stages of blood-stage parasites (P2 and P3 compartment) based on previously published information (21, 22). The implementation of the drug effect was described by the following differential equations (equations 14 and 15). Cure was assumed to be achieved when the total parasite biomass (P1 + P2 + P3) was less than 1 parasite, triggering the PMR_LC_ to be 1 (i.e., resulting in no parasite growth).

(14) $$\frac{d\text{P}2}{\mathrm{dt}}=\text{P}1\times k_{\mathrm{MAT}}\times \mathrm{RE}{\text{G}}_{1}-\text{P}2\times k_{\mathrm{SQ}}\times \mathrm{RE}{\text{G}}_{2}-\text{P}2\times k_{\mathrm{RUP}}\times \mathrm{RE}{\text{G}}_{3}-\text{P}2\times \text{EFF}$$

(15) $$\frac{d\text{P}3}{\mathrm{dt}}=\text{P}2\times k_{\mathrm{SQ}}\times \mathrm{RE}{\text{G}}_{2}-\text{P}3\times k_{\mathrm{RUP}}\times \mathrm{RE}{\text{G}}_{3}-\text{P}3\times \text{EFF}$$

The simulation-based diagnostics (i.e., visual predictive checks) of the final pharmacokinetic-pharmacodynamic model was based on 1,000 simulations using the individual parameter estimates from the final pharmacokinetic model and the final parameter estimates from the pharmacokinetic-pharmacodynamic model. Frequent dummy time points were added to the data set for simulations (every 5 h, from 0 h to 576 h). The visual predictive checks were stratified on the day of piperaquine treatment and the predicted treatment outcome (curative versus recrudescent infection). The observed data were overlaid with the 90% prediction interval from 1,000 simulations to evaluate the predictive performance of the model.

### Clinical scenario simulations.

The final pharmacokinetic-pharmacodynamic model describing the dynamic parasite growth and piperaquine *in vivo* parasiticidal effects was used to perform population-based simulations of clinical scenarios in NONMEM. Simulations were performed to predict the probability of treatment failure at different levels of drug resistance. The total number of circulating parasites was initialized with 10^6^ or 10^10^ parasites in order to simulate asymptomatic and symptomatic infections, respectively. The following six different clinical scenarios with resistant infections were simulated to predict the probability of treatment failure: (1) absence of artemisinin resistance and absence of piperaquine resistance, (2) presence of artemisinin resistance and absence of piperaquine resistance, (3) absence of artemisinin resistance and presence of piperaquine resistance (2 × EC_50_ of piperaquine), and (4) to (6) presence of artemisinin resistance and presence of piperaquine resistance (2 × EC_50_, 3 × EC_50_, and 4 × EC_50_ of piperaquine).

Dihydroartemisinin was used as a representative of the artemisinin derivative, and pharmacokinetic parameters of dihydroartemisinin were taken from a previously published population pharmacokinetic analysis (42). The parasiticidal effect of dihydroartemisinin was adjusted based on the observed parasite clearance half-life from the Tracking Resistance to Artemisinin Collaboration (TRAC) study data (28). The reported mean parasite clearance half-life for sensitive (2.5 h) and resistant (6.2 h) infections from the TRAC study was used to calculate the parasite clearance slope and generate parasite clearance profiles, starting at an initial total circulating parasite density of 10^11^ parasites (Fig. S5). Simulations were performed in Berkeley Madonna (43) using the developed semimechanistic growth model to adjust the *E*_max_ values of dihydroartemisinin to match the parasite clearance profiles generated from the parasite clearance half-life reported in the TRAC study. The derived adjusted *E*_max_ values, resulting in equivalent residual total circulating parasites at 72 h as seen in the TRAC study, were used throughout the simulations.

The parasite killing effect of dihydroartemisinin was implemented in all parasite compartments (P1, P2, and P3) using an *E*_max_ function (equation 13) because dihydroartemisinin has an effect on almost all stages of the parasite life cycle (44). Treatment failure was defined as a predicted total parasite biomass of >1 parasites at 30 days after the first dose of piperaquine was given. Additionally, simulations were conducted to predict the probability of treatment failure when adding an additional hypothetical drug to the conventional dihydroartemisinin-piperaquine regimen. The effect of the hypothetical drug was implemented in the same manner as the parasite killing effect of piperaquine (added on P2 and P3 compartments). Different efficacy and duration of action of the hypothetical drug were investigated, including drugs demonstrating a PRR_LC_ of 10^1^, 10^2^, and 10^3^ and a duration of action of 1 week, 2 weeks, 3 weeks, 4 weeks, and 5 weeks. The following differential equations described the drug effects for the simulations (equation 16 to 18)

(16) $$\frac{d\text{P}1}{\mathrm{dt}}=-\text{P}1\times k_{\mathrm{MAT}}\times \mathrm{RE}{\text{G}}_{1}+\left(\text{P}2+\text{P}3\right)\times k_{\mathrm{RUP}}\times \mathrm{RE}{\text{G}}_{3}\times \mathrm{PM}{\text{R}}_{\mathrm{LC}}-\text{P}1\times \text{EF}{\text{F}}_{1}$$

(17) $$\frac{d\text{P}2}{\mathrm{dt}}=\text{P}1\times k_{\mathrm{MAT}}\times \mathrm{RE}{\text{G}}_{1}-\text{P}2\times k_{\mathrm{SQ}}\times \mathrm{RE}{\text{G}}_{2}-\text{P}2\times k_{\mathrm{RUP}}\times \mathrm{RE}{\text{G}}_{3}-\text{P}2\times \text{EF}{\text{F}}_{1}-\text{P}2\times \text{EF}{\text{F}}_{2}-\text{P}2\times \text{EF}{\text{F}}_{3}$$

(18) $$\frac{d\text{P}3}{\mathrm{dt}}=\text{P}2\times k_{\mathrm{SQ}}\times \mathrm{RE}{\text{G}}_{2}-\text{P}3\times k_{\mathrm{RUP}}\times \mathrm{RE}{\text{G}}_{3}-\text{P}3\times \text{EF}{\text{F}}_{1}-\text{P}3\times \text{EF}{\text{F}}_{2}-\text{P}3\times \text{EF}{\text{F}}_{3}$$

where EFF_1_ denotes the parasiticidal effect of dihydroartemisinin, EFF_2_ denotes the parasiticidal effect of piperaquine, and EFF_3_ denotes the parasiticidal effect of the third hypothetical drug.

Each simulation scenario consisted of 4,800 simulated patients, including 48 individuals with various times of first dose with a 1-h difference among each simulated patient from 0 to 48 h (100 simulations). The variation mimics a real-life scenario where patients present to the clinic and receive treatment at different stages of the parasite life cycle. The standard 3-day dose of dihydroartemisinin-piperaquine (120/960 mg) for a patient weighing 60 kg was used in all simulations. A schematic illustration of the structural model used for simulations is presented in the supplemental material (Fig. S7).

## Supplementary Material

Supplemental file 1
