# Supplementary material for: Semimechanistic Pharmacokinetic and Pharmacodynamic Modeling of Piperaquine in a Volunteer Infection Study with Plasmodium falciparum Blood-Stage Malaria
Source: Antimicrob Agents Chemother. 2021 Mar 18;65(4):e01583-20. doi: 10.1128/AAC.01583-20 (PMC8097471; doi:10.1128/AAC.01583-20)
Supplement: Supplemental file 1 [file AAC.01583-20-s0001.pdf]

SUPPLEMENTAL MATERIAL

Wattanakul et al. Antimicrobial Agents and Chemotherapy. April 2021 Volume 65 Issue 4 e01583-20.

TABLE S1 Log-linear growth model parameter estimates

Parameter	Population estimate * (% RSE)	95% CI	IIV * (% RSE)	95% CI	Shrinkage (%)
**F_{SUR} (%)**	5 fixed	-	-	-	-
**k_G (h^{-1})**	0.0661 (5.70)	0.0631–0.0688	20.1 (25.6)	1.38–28.7	27.3
**T_{PC} (h)**	38.8 fixed	-	-	-	-
**σ**	3.68 (15.9)	2.53–4.82	-	-	24.5

* Population mean parameters estimated from NONMEM. Inter-individual variability (IIV) are presented as the coefficient of variation (%CV), calculated as $100\times\sqrt{\exp(\text{estimate})-1}$.

k_G, parasite growth rate (h^{-1}); T_{PC}, duration of the parasite life cycle; and σ, residual unexplained variability.

TABLE S2 Sine-wave growth model parameter estimates

Parameter	Population estimate * (% RSE)	95% CI	IIV * (% RSE)	95% CI	Shrinkage (%)
**a (intercept)**	4.50 fixed	-	-	-	-
**k_G (h^{-1})**	0.0655 (2.30)	0.0630–0.0680	9.18 (21.2)	3.67–12.5	15.4
**c (amplitude)**	1.68 (15.1)	1.18–2.18	-	-	-
**k (phase shift)**	6.91 (1.30)	6.73–7.09	-	-	-
**T_{PC} (h)**	38.8 fixed	-	-	-	-
**σ**	2.22 (23.4)	1.20–3.24	-	-	26.7

* Population mean parameters estimated from NONMEM. Inter-individual variability (IIV) are presented as the coefficient of variation (%CV), calculated as $100\times\sqrt{\exp(\text{estimate})-1}$.

a, y-intercept (i.e. the P_{CIR} at time zero); c, sine-wave amplitude; T_{PC}, duration of the parasite life cycle (fixed to 38.8 h); k, sine-wave phase shift; and σ, residual unexplained variability.

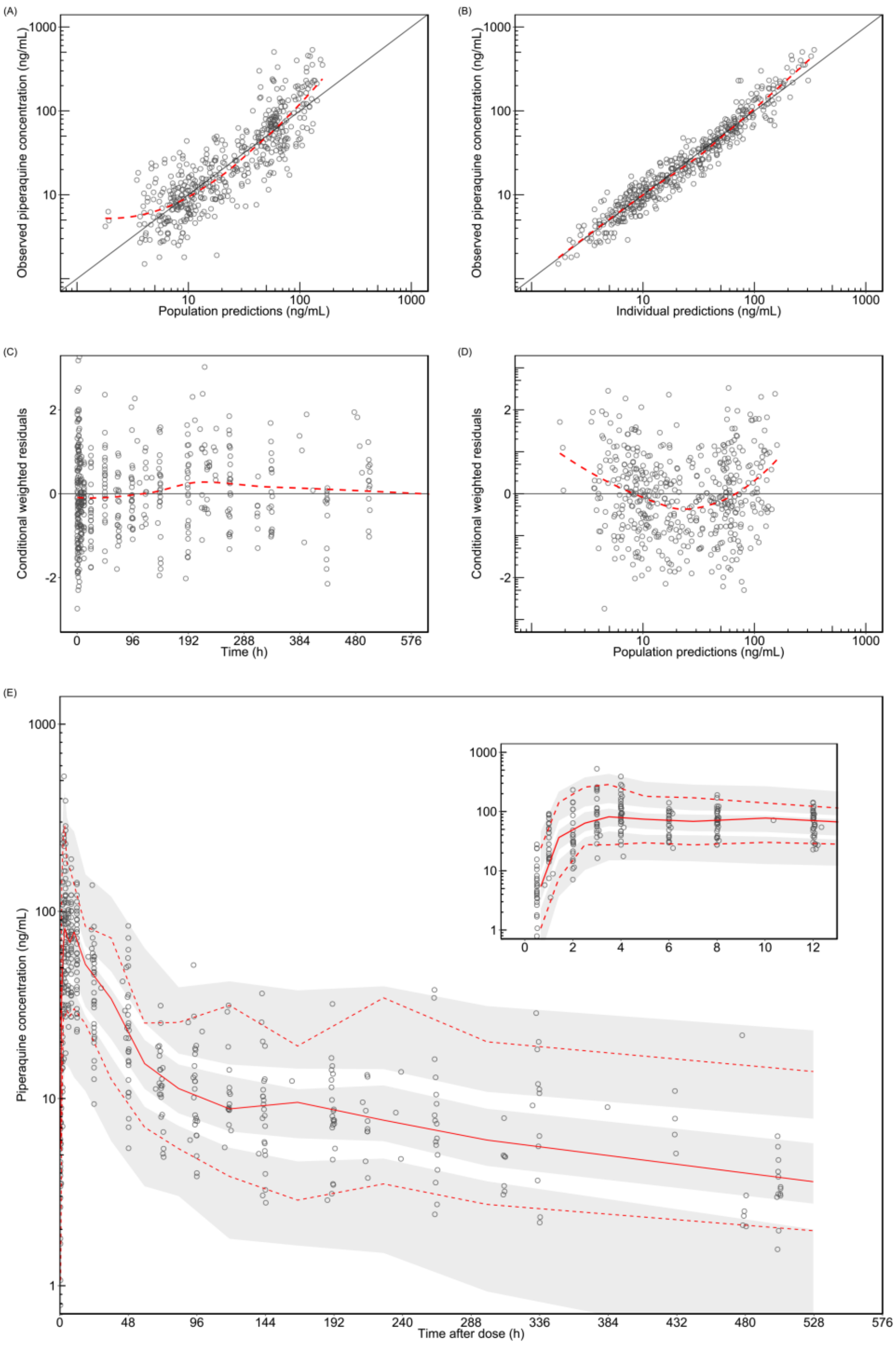

FIG S1 Model diagnostics of the final piperaquine pharmacokinetic model.
Goodness-of-fit plots (A-D); observed piperaquine concentrations versus population predictions (A), observed piperaquine concentrations versus individually predicted concentrations (B), conditionally weighted residual versus time after dose (C), and conditionally weighted residual versus population predictions (D). The open circles represent the observed piperaquine concentrations. The solid black lines represent the line of identity and the dashed red lines represent a local polynomial regression fitting of all observations. Visual predictive check (n = 2,000) (E). The open circles represent the observed piperaquine concentrations. Solid red lines represent the 50th percentiles of the observations, and dashed red lines represent the 5th and 95th percentiles of the observations. The shaded areas represent the 95% confidence intervals of each simulated percentile. The insert shows the first 12 hours after treatment.

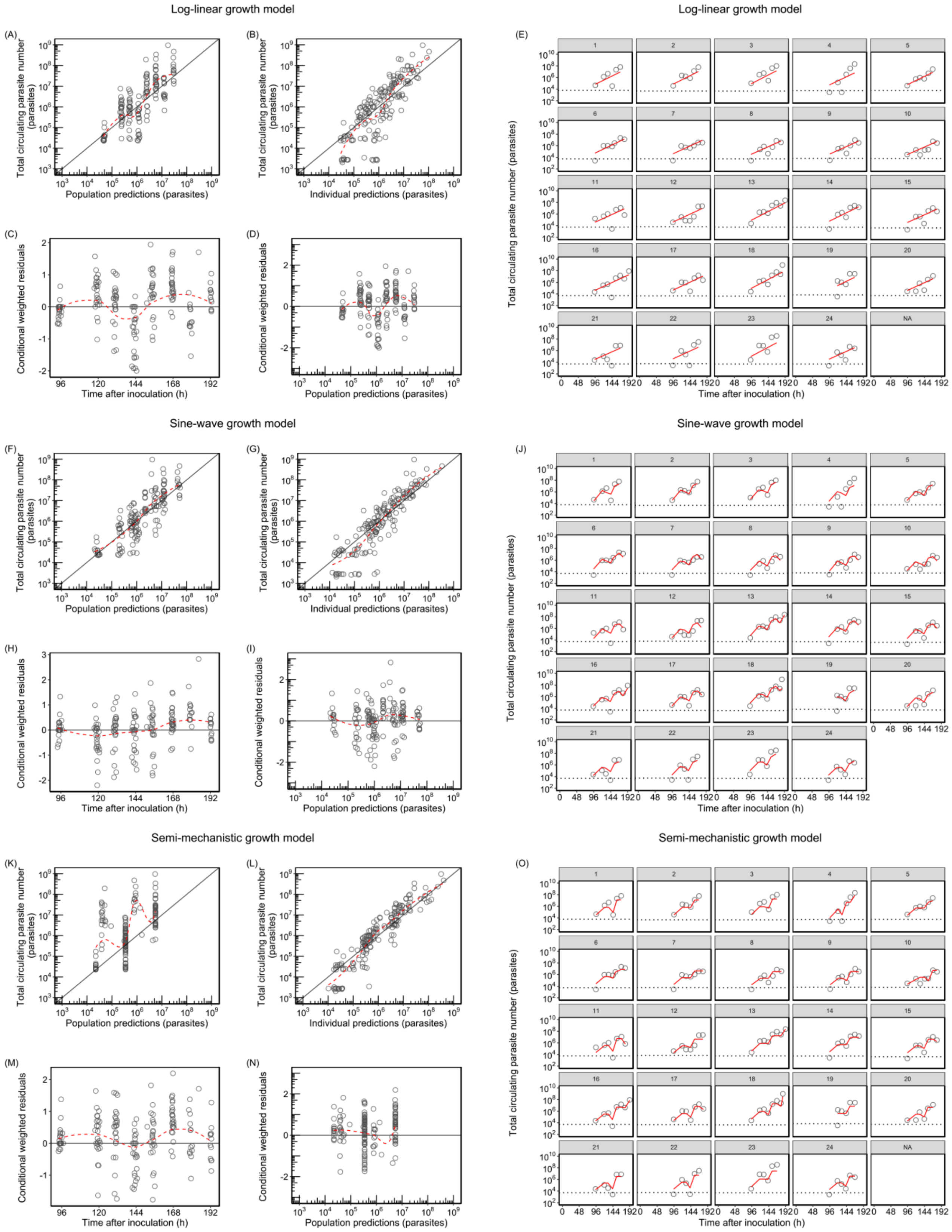

FIG S2 Goodness-of-fit and individual plots of the investigated growth model.
The upper panel, middle panel, and lower panel show the goodness-of-fit plots and individual plots of the log-linear growth model, sine-wave growth model, and the semi-mechanistic growth model, respectively, using the growth phase parasite data only.

Wattanakul et al. Supplemental Material.

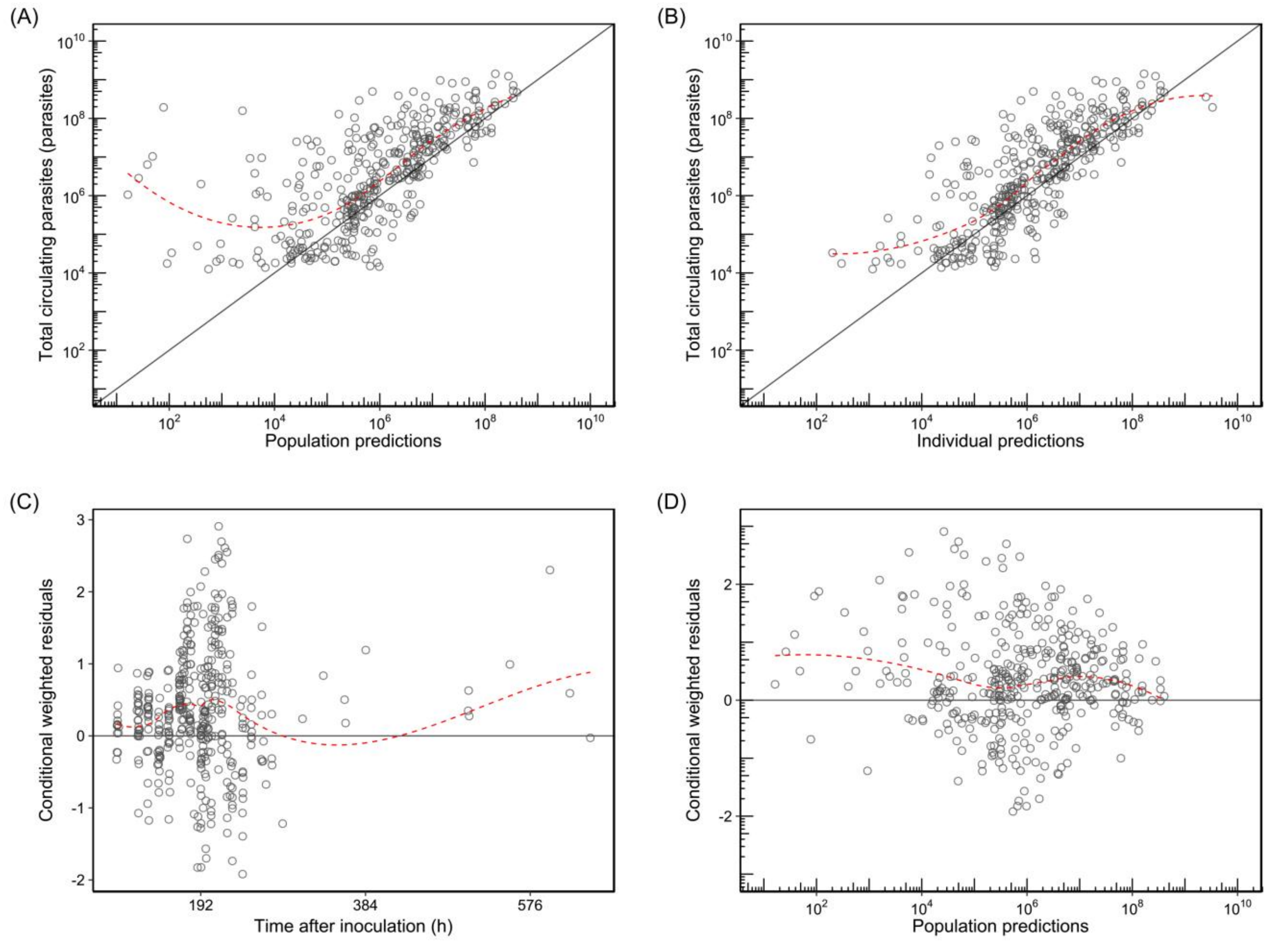

FIG S3 Goodness-of-fit of the final population pharmacokinetic-pharmacodynamic model.
Total circulating parasites versus population predictions (A). Total circulating parasites versus individual predictions (B). Conditionally weighted residual versus time after dose (C). Conditionally weighted residual versus population predictions (D). The solid black lines represent the line of identity and the dashed red lines represent a local polynomial regression fitting of all observations.

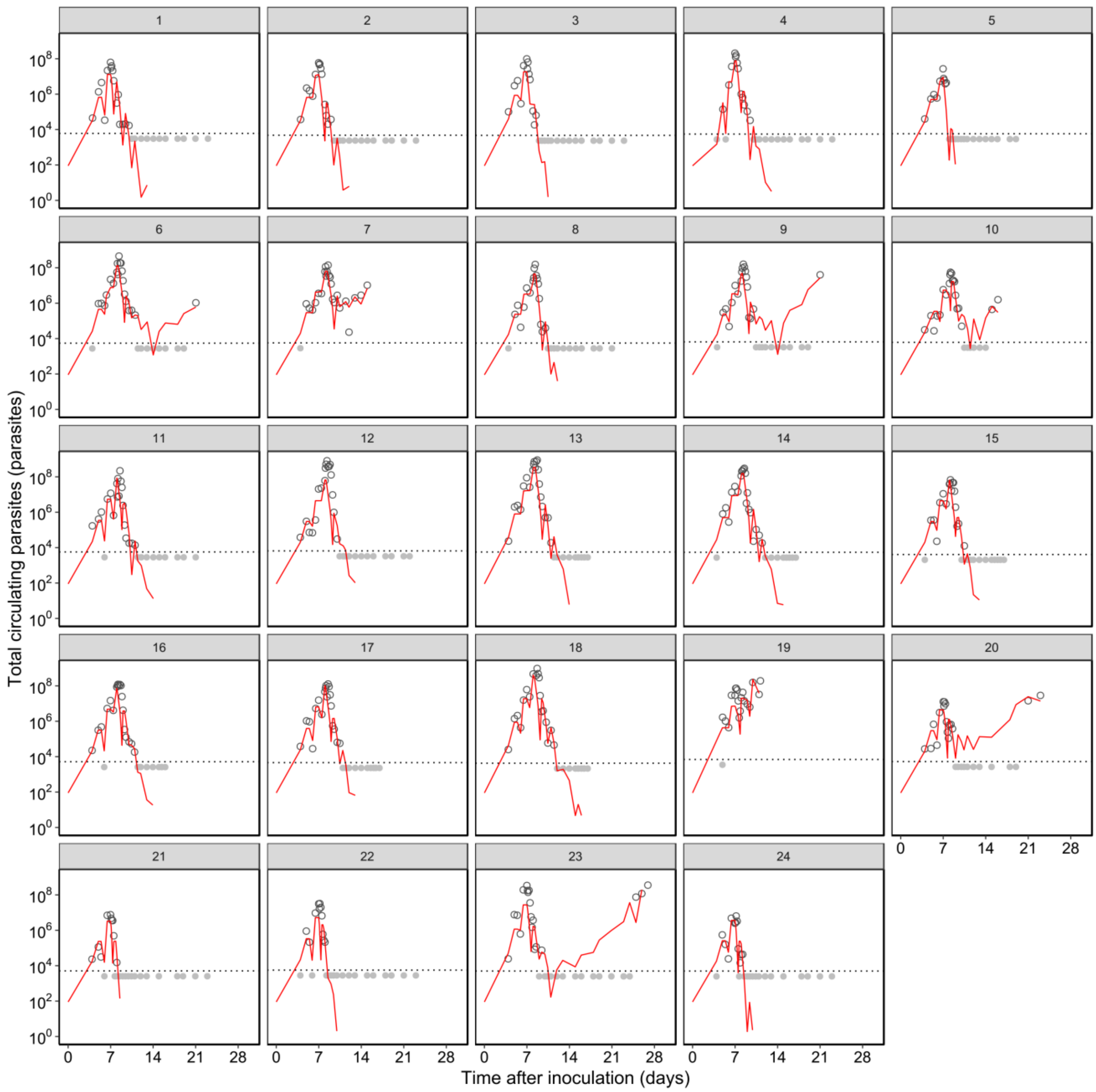

FIG S4 Individual fits of the final pharmacokinetic-pharmacodynamic model.
The open circles represent the observed total circulating parasites, the red line represents the individual prediction from the model, and the horizontal dotted line represents the individual lower limit of parasite detection (LOD). Measured parasite density below LOD is marked in the graph as filled circles.

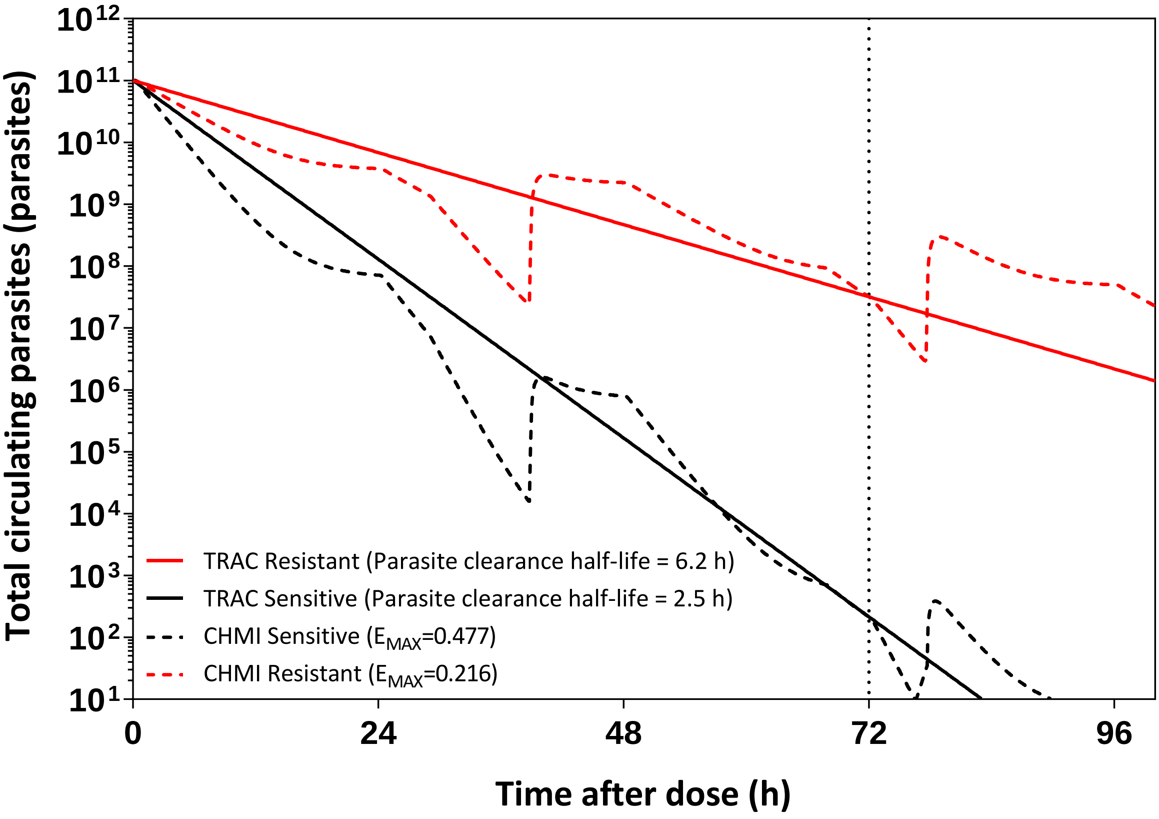

FIG S5 Simulated parasiticidal activity.

Simulated parasiticidal activity of dihydroartemisinin from the developed semi-mechanistic model, overlaid with the parasiticidal activity of artesunate based on *in vivo* parasite clearance half-lives. The solid lines represent the parasite killing capacity of artesunate from the Tracking Resistance to Artemisinin Collaboration (TRAC) study (black solid line, sensitive infection; red solid line, resistant infection). The dashed lines represent the adjusted parasite killing capacity of dihydroartemisinin using the semi-mechanistic model (black dashed line, sensitive infection; red dashed line, resistant infection).

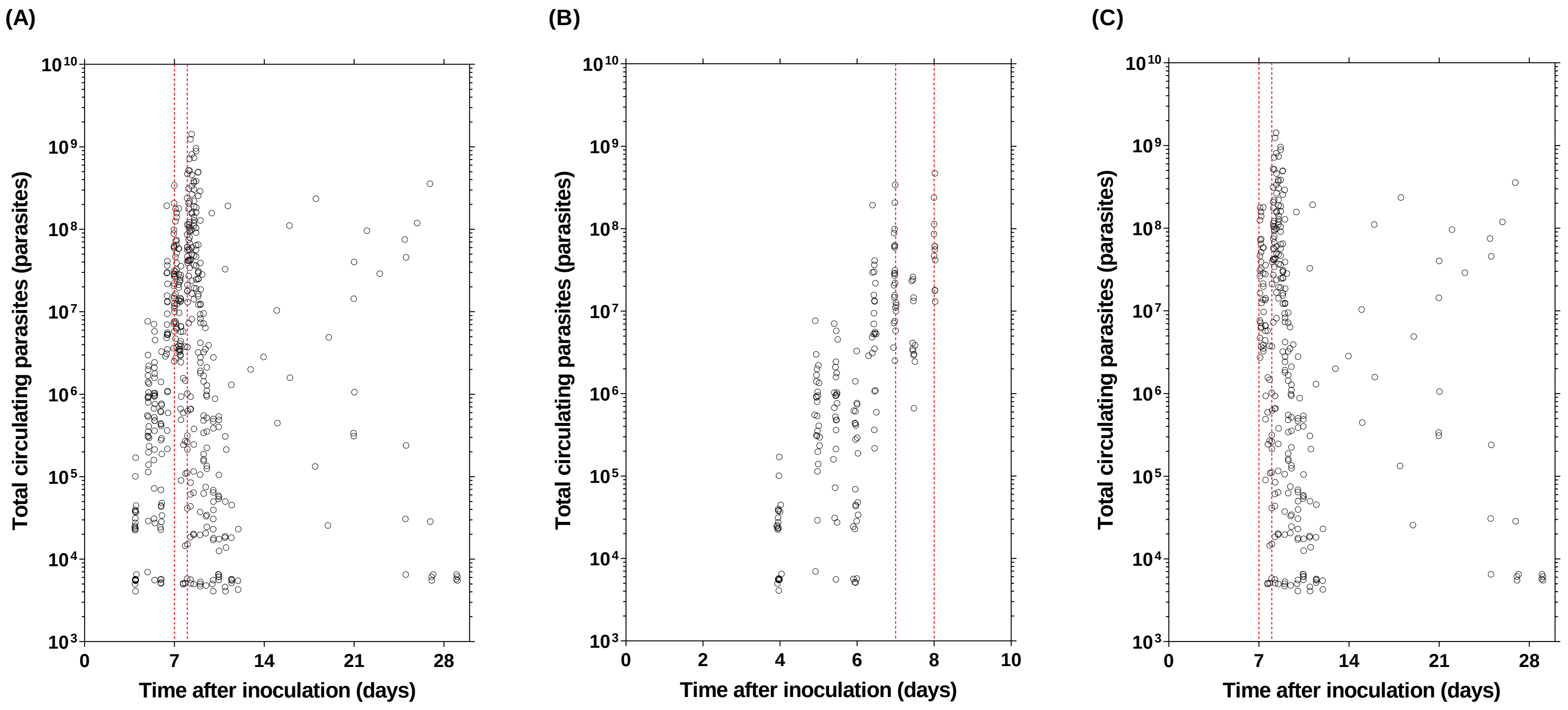

FIG S6. Scatter plots of the observed total circulating parasites versus time after inoculation (days) from the IBSM study.
All data used for pharmacokinetic-pharmacodynamic modelling (A). Data during the growth phase (B). Data during the elimination phase after the administration of piperaquine, and the recrudescence of the parasites after piperaquine is eliminated (C). The dotted red lines represent the time when the first dose of piperaquine was given (day 7, n = 11 or day 8, n = 13).

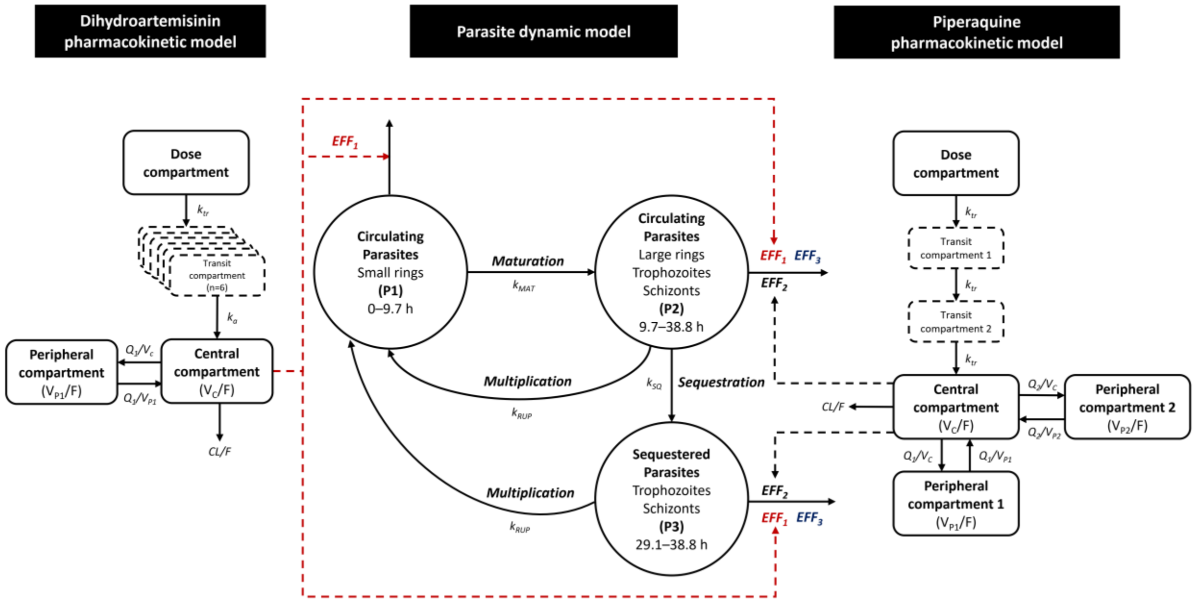

FIG S7 Schematic illustration of the pharmacokinetic-pharmacodynamic model used for simulations. Schematic of the pharmacokinetic-pharmacodynamic model used for simulations, incorporating the effect of dihydroartemisinin, piperaquine, and the third hypothetical drug used for simulation of clinical scenarios. In the dihydroartemisinin pharmacokinetic model (left) and piperaquine pharmacokinetic model (right), F represents relative bioavailability, k_{tr} represents transit rate constant, k_a represents first-order rate constant of absorption, CL/F represents apparent oral clearance, V_C/F represents apparent central volume of distribution, Q/F represents inter-compartmental clearance from central compartment to peripheral compartment, and V_P/F represents apparent peripheral volume of distribution. In the parasite dynamic model (middle), k_{MAT} represents first-order rate constant of parasite maturation, k_{SQ} represents first-order rate constant of parasite sequestration, and k_{RUP} represents first-order rate constant of schizont rupture. The parasite killing effect of each drug (EFF) was described by an E_{max} function, where EFF_1 represents the parasite killing effect of dihydroartemisinin, EFF_2 represents the parasite killing effect of piperaquine, and EFF_3 represents the parasite killing effect of the third hypothetical drug.

NONMEM code

```
$PROBLEM    PK/PD OF PIPERAQUINE IN INDUCED BLOOD-STAGE MODEL

$INPUT
ID                          ; PARTICIPANT ID
TIME                        ; TIME (H)
TAD                         ; TIME AFTER DOSE (H)
AMT                         ; DOSE AMOUNT OF PIPERAQUINE (MG)
EVID                        ; EVENT ID RECORD
CMT                         ; COMPARTMENT
BQL                         ; DATA BELOW LOD (1=YES)
ILOD                        ; INDIVIDUAL LOWER LIMIT OF DETECTION (1×BW×80)
DV                          ; DEPENDENT VARIABLE (OBSERVED PARASITAEMIA)
MDV                         ; MISSING VARIABLE
IF1                         ; INDIVIDUAL RELATIVE BIOAVAILABILITY
IMT                         ; INDIVIDUAL MEAN TRANSIT TIME
ICL                         ; INDIVIDUAL CLEARANCE
IV2                         ; INDIVIDUAL CENTRAL VOLUME OF DISTRIBUTION
IQ1                         ; INDIVIDUAL INTER-COMPARTMENT CLEARANCE (V2 AND V3)
IV3                         ; INDIVIDUAL FIRST PERIPHERAL VOLUME OF DISTRIBUTION
IQ2                         ; INDIVIDUAL INTER-COMPARTMENT CLEARANCE (V3 AND V4)
IV4                         ; INDIVIDUAL SECOND PERIPHERAL VOLUME OF DISTRIBUTION

$DATA
PKPD_dataset_PQP_CHMI.csv IGNORE= @

$SUBROUTINE
ADVAN13 TOL= 3 ; SPECIFY SUBROUTINE

$MODEL
NCOMP=9                     ; NO. OF COMPARTMENT
COMP= (1)                   ; ABSORPTION COMPARTMENT
COMP= (2)                   ; CENTRAL COMPARTMENT
COMP= (3)                   ; PERIPHERAL COMPARTMENT 1
COMP= (4)                   ; PERIPHERAL COMPARTMENT 2
COMP= (5)                   ; TRANSIT COMPARTMENT 1
COMP= (6)                   ; TRANSIT COMPARTMENT 2
COMP= (7)                   ; VISIBLE PARASITE COMPARTMENT 1 (SMALL RING)
COMP= (8)                   ; VISIBLE PARASITE COMPARTMENT 2 (LARGE RING, TROPH, SCHIZONT)
COMP= (9)                   ; SQ COMPARTMENT (TROPH, SCHIZONT)

$PK
; VERBATIM-CODE
"FIRST
" COMMON /PRCOMG/
IDUM1,IDUM2,IMAX,IDUM4,IDUM5
" INTEGER IDUM1,IDUM2,IMAX,IDUM4,IDUM5
" IMAX=1000000

; IPK-FROM-FINAL-PK-MODEL
F1= IF1                     ; INDIVIDUAL RELATIVE BIOAVAILABILITY
MT= IMT                     ; INDIVIDUAL MEAN TRANSIT TIME
CL= ICL                     ; INDIVIDUAL PIPERAQUINE APPARENT CLEARANCE
V2= IV2                     ; INDIVIDUAL CENTRAL VOLUME OF DISTIBUTION
Q1= IQ1                     ; INDIVIDUAL INTERCOMPARTMENTAL CLEARANCE
V3= IV3                     ; INDIVIDUAL PERIPHERAL VOLUME OF DISTRIBUTION
Q2= IQ2                     ; INDIVIDUAL INTERCOMPARTMENTAL CLEARANCE
```

```
V4= IV4                                    ; INDIVIDUAL PERIPHERAL VOLUME OF DISTRIBUTION
NN= 2                                      ; NUMBER OF TRANSIT COMPARTMENT
KTR= (NN+1)/MT                             ; TRANSIT RATE CONSTANT
S2= V2/1000                                ; SCALING FACTOR
K15= KTR                                   ; RATE CONSTANT FOR DIFFERENTIAL EQUATION
K56= KTR                                   ; RATE CONSTANT FOR DIFFERENTIAL EQUATION
K62= KTR                                   ; RATE CONSTANT FOR DIFFERENTIAL EQUATION
K23= Q1/V2                                 ; RATE CONSTANT FOR DIFFERENTIAL EQUATION
K32= Q1/V3                                 ; RATE CONSTANT FOR DIFFERENTIAL EQUATION
K24= Q2/V2                                 ; RATE CONSTANT FOR DIFFERENTIAL EQUATION
K42= Q2/V4                                 ; RATE CONSTANT FOR DIFFERENTIAL EQUATION
K20= CL/V2                                 ; RATE CONSTANT FOR DIFFERENTIAL EQUATION

; PKPD-MODEL
FSURV= THETA(1)*EXP(ETA(1))                ; PARASITE SURVIVAL AFTER INOCULATION
A_0(7)= 1800*FSURV                         ; INITIALIZED P1 WITH INITAIL PARASITE NUMBER (1800)
A_0(8)= 0                                  ; INITAILIZED P2
A_0(9)= 0                                  ; INITAILIZED P3
CYCLE= THETA(2)*EXP(ETA(2))                ; PARASITE LIFE CYCLE TIME (HOURS)
K78= THETA(3)*EXP(ETA(3))                  ; TRANSFER RATE FROM P1 TO P2
TSEQ= THETA(4)+(ETA(4))                    ; LAG TIME OF SEQUESTRATION (DELAYED FROM 19.4HR)
FSQ= THETA(5)*EXP(ETA(5))                  ; FRACTION OF PARASITE SEQUESTRATION
KSQ= LOG(100/(100-FSQ))/(CYCLE-MMI2)       ; PARASITE SEQUESTRATION RATE
KR= THETA(6)*EXP(ETA(6))                   ; BURST FROM SEQUESTERED - RETURN TO VISIBLE
FOLD= THETA(7)*EXP(ETA(7))                 ; MULTIPLICATION RATE (FOLD INCREASED)

; DRUG-EFFECT
EMAX= THETA(8)*EXP(ETA(8))                 ; EMAX – PARASITE KILLING RATE
EC50= THETA(9)*EXP(ETA(9))                 ; EC50
SHP= THETA(10)*EXP(ETA(10))                ; SHAPE OF EMAX CURVE

$DES
;PK-MODEL
DADT(1)= - A(1)*K15
DADT(2)= A(6)*K62 + A(3)*K32 + A(4)*K42 - A(2)*K23 - A(2)*K24 - A(2)*K20
DADT(3)= A(2)*K23 - A(3)*K32
DADT(4)= A(2)*K24 - A(4)*K42
DADT(5)= A(1)*K15 - A(5)*K56
DADT(6)= A(5)*K56 - A(6)*K62

; SQUARE-WAVE  FOR  PARASITE MOVEMENT
PI = 3.14159265359

; REG1(CREN1)
MMI1=(36/48)*CYCLE                         ; SHIFT (HOW LONG TO SPEND IN STATE 0)
PSH1=2*PI                                  ; PEAK SHIFT
PER1=2*PI*MMI1/CYCLE                       ; PERIOD
SI1=SIN((PI-PER1)/2)                       ; SINEWAVE-1
SI2=SIN(2*PI*T/CYCLE+(PI-PER1+PSH1)/2)     ; SINEWAVE-2
CREN1=(SQRT(SI2-SI1)*(SI2-SI1)))-(SI2-SI1))/(2*SQRT(10E-6 +((SI2-SI1)*(SI2-SI1))))   ;REG1

; REG2(CREN2)
MMI2=((24/48)*CYCLE) + TSEQ                ; SHIFT (HOW LONG TO SPEND IN STATE 0)
PSH2=0                                     ; PEAK SHIFT
PER2=2*PI*MMI2/CYCLE                       ; PERIOD
SI3=SIN((PI-PER2)/2)                       ; SINEWAVE-3
SI4=SIN(2*PI*T/CYCLE+(PI-PER2+PSH2)/2)     ; SINEWAVE-4
```

```
CREN2=(SQRT( ((SI4-SI3)*(SI4-SI3)))-(SI4-SI3))/(2*SQRT(10E-6 +((SI4-SI3)*(SI4-SI3))))   ; REG2

; REG3(CREN3)
MMI3=(38/48)*CYCLE                          ; SHIFT (HOW LONG TO SPEND IN STATE 0)
PSH3=3.16*PI                                ; PEAK SHIFT
PER3=2*PI*MMI3/CYCLE                        ; PERIOD
SI5=SIN((PI-PER3)/2)                        ; SINEWAVE-5
SI6=SIN(2*PI*T/CYCLE+(PI-PER3+PSH3)/2)      ; SINEWAVE-6
CREN3=(SQRT( ((SI6-SI5)*(SI6-SI5)))-(SI6-SI5))/(2*SQRT(10E-6 +((SI6-SI5)*(SI6-SI5))))   ; REG3

; PIPERAQUINE-EFFECT
CP=A(2)/S2                                  ; PIPERAQUINE CONCENTRATION (NG/ML)
DELCP=1E-6                                  ; AVOID ZERO
EFF=EMAX*(((CP+DELCP)**SHP)/(((CP+DELCP)**SHP)+((EC50+DELCP)**SHP)))  ;PIPERAQUINE EFFECT

; PD-MODEL
APARA= A(7)+A(8)+A(9)                       ; TOTAL PARASITEMIA
IF (APARA.LT.1) THEN
REC1= 1/FOLD                                ; FOLD.EQ.1
ELSE
REC1= 1                                     ; FOLD.EQ.FOLD
ENDIF

DADT (7)= - A(7)*K78*CREN1 + A(8)*KR*CREN3*FOLD*REC1 + A(9)*KR*CREN3*FOLD*REC1
DADT (8)= - A(8)*EFF + A(7)*K78*CREN1 - A(8)*KSQ*CREN2 - A(8)*KR*CREN3
DADT (9)= - A(9)*EFF + A(8)*KSQ*CREN2 - A(9)*KR*CREN3

$ERROR
DEL= 1E-12
VISIBLE= A(7)+ A(8)
TPARA= A(7)+ A(8)+ A(9)
IPRED= LOG(VISIBLE+DEL)
W=SQRT(SIGMA(1,1))
IF(W.EQ.0) W = 1
IRES= IPRED-DV
IWRES= (IPRED-DV)/W

; M3 METHOD
Y= IPRED+EPS(1)
IF (CMT.EQ.7) LLOQ = LOG(ILOD)              ; INDIVIDUAL LOD CALCULATED FROM 1*80*BW
DUM  = (LLOQ-IPRED)/W                       ; POSITIVE WHEN IPRED IS LARGER THEN LLOQ
CUMD = PHI(DUM)

;PREDICTION
IRES  = IPRED-DV
IWRES = IRES/W
IF(BQL.EQ.0.AND.CMT.EQ.7) THEN
F_FLAG = 0
Y = IPRED+ERR(1)
ENDIF

; LIKELIHOOD DV<LOD
IF(BQL.EQ.1) THEN
F_FLAG = 1
Y     = CUMD + DEL
MDVRES = 1
ENDIF
```

```
$THETA
(0.05) FIX                          ; 1. FSURV
(38.8) FIX                          ; 2. CYCLE
(2) FIX                             ; 3. K78
(9.74) FIX                          ; 4. TSEQ
(90) FIX                            ; 5. FSQ
(2) FIX                             ; 6. KR
(15.7) FIX                          ; 7. FOLD
(0, 0.3, 1)                         ; 8. EMAX
(0, 10)                             ; 9. EC50
(2.8) FIX                           ; 10. SHP

$OMEGA 0 FIX                        ; 1. IIV_FSURV
$OMEGA 0.00359 FIX                  ; 2. IIV_CT
$OMEGA 0 FIX                        ; 3. IIV_K78
$OMEGA 0 FIX                        ; 4. IIV_TSEQ
$OMEGA 0 FIX                        ; 5. IIV_FSQ
$OMEGA 0 FIX                        ; 6. IIV_KR
$OMEGA 0.0328 FIX                   ; 7. IIV_FOLD
$OMEGA 0.01                         ; 8. IIV_EMAX
$OMEGA 0.01                         ; 9. IIV_EC50
$OMEGA 0 FIX                        ; 10. IIV_SHP

$SIGMA 4.76                         ; 1. Additive ERR

$ESTIMATION
MAXEVAL=9999 PRINT=1 METHOD=1 LAPLACIAN NUMERICAL SLOW INTER POSTHOC NSIG=1 SIGL=3
SADDLE_RESET=1 SADDLE_HESS=1 MCETA=100 RANMETHOD=P NOABORT

$COVARIANCE PRINT=E
```
